# Independent proviral and antiviral host factors recognize the same capsid protein in divergent human herpesviruses

**DOI:** 10.1371/journal.ppat.1014376

**Published:** 2026-07-08

**Authors:** Yiqi Zhao, Sarah Probert, Jan Birkel, Xin Liu, Yaling Shi, Olivia Shulan Yang, Kangyan Zhao, Yongxu Lu, Geoffrey L. Smith

**Affiliations:** 1 Sir William Dunn School of Pathology, University of Oxford, Oxford, United Kingdom; 2 Chinese Academy of Medical Sciences–Oxford Institute, University of Oxford, Oxford, United Kingdom; 3 Shanghai Institute of Materia Medica, Chinese Academy of Sciences, Shanghai, P.R. China; Cardiff University, UNITED KINGDOM OF GREAT BRITAIN AND NORTHERN IRELAND

## Abstract

Intrinsic cellular factors that inhibit herpesvirus infection remain incompletely defined. Here, we identify TRIM5α as a restriction factor for herpes simplex virus type 1 (HSV-1). TRIM5α-mediated restriction requires its ubiquitin ligase activity, PRY–SPRY domain, and the ability to oligomerize. Mechanistically, we show that TRIM5α directly engages capsid protein VP19C and promotes the stability of the VP19C–VP23 complex and its nuclear accumulation. VP19C also activates NF-κB synergistically with TRIM5α and independently. HSV-1 counteracts this host defense by triggering proteasome-dependent TRIM5α degradation. In addition, we show that Cyclophilin A (CypA), which is incorporated into HSV-1 virions, also binds to VP19C, but enhances infection. As with HIV-1 and orthopoxviruses, the proviral activity of CypA is disrupted by cyclosporin A (CsA), but unlike the situation with these other viruses, the proviral activity of CypA is independent of TRIM5α. Notably, CsA and its non-immunosuppressive derivatives also exhibit anti-HSV-1 activity in neuronal cell lines, suggesting a potential therapy for HSV-1 encephalitis. TRIM5α and CypA also interact with orthologs of VP19C in other alpha, beta and gamma human herpesviruses. These findings reveal two distinct host pathways acting on the herpesvirus capsid and provide a foundation for comparing how TRIM5α and CypA modulate infection of unrelated virus families, offering new directions to identify shared principles of host recognition and viral evasion.

## Author summary

Herpes simplex virus type 1 (HSV-1) is an important human pathogen that establishes lifelong infection, yet how cells sense and restrict HSV-1 infection and how HSV-1 escapes restriction remain poorly defined. This study shows that two human proteins, TRIM5α and Cyclophilin A (CypA), engage the same HSV-1 capsid protein but drive opposing outcomes: TRIM5α restricts infection, whereas CypA enhances it. Notably, cyclosporin A (CsA), which targets CypA, exhibits antiviral activity in neuronal cell lines, suggesting that its non-immunosuppressive derivatives may be potential antivirals in treating HSV-1–associated encephalitis. The ability of TRIM5α and CypA to recognize the same capsid protein from divergent human herpesviruses indicates that these capsids share structural features that are sensed by host factors. By defining these interactions, this work illustrates how host proteins modulate diverse herpesviruses and suggests avenues for broad-spectrum antiviral design.

## Introduction

Human herpesviruses impose a substantial global health burden because they establish lifelong latency and periodically reactivate to cause disease. HSV-1 is a highly prevalent human pathogen, infecting 67% of humans under 50 years old [1], and its clinical manifestations range from oral and genital ulcerations to keratitis, conjunctivitis, and life-threatening encephalitis. Neonates and immunocompromised patients are particularly vulnerable to severe complications [2]. Despite decades of effort, no licensed HSV-1 vaccine exists, and current antiviral therapies do not eliminate latent infection. Moreover, antiviral resistance is increasingly observed in immunocompromised populations [3,4]. These limitations highlight the need for new antiviral strategies with different targets that are less susceptible to the emergence of drug resistance. Host factors that promote viral replication are attractive drug targets because viruses cannot readily mutate to escape. Understanding HSV-1–host interactions at the molecular level is therefore essential for identifying host factors that could be targeted by antiviral drugs.

Viral capsids are promising targets for pattern recognition receptors (PRRs) because they are ordered, repeating structures whose patterns can be distinguished from host proteins. HSV-1 VP19C is a minor capsid protein that forms a heterotrimer with two VP23 molecules [5]. Together, they stabilize the major capsid protein VP5 in the assembled virion [5,6]. Prior to capsid assembly in the nucleus, VP19C interacts with VP23 and VP5 in the cytoplasm and mediates their nuclear import [7,8]. The N terminus of VP19C is flexible due to its high glycine content and contains a nuclear localization signal (NLS) [9]. The C terminus is structurally less flexible and contains a nuclear export signal (NES) that, together with the NLS, allow nucleocytoplasmic shuttling [10]. Orthologs of HSV-1 VP19C are present in other human herpesviruses such as HSV-2 VP19C, varicella-zoster virus (VZV) ORF20 [11], human cytomegalovirus (HCMV) UL46 [12], Kaposi’s sarcoma-associated herpesvirus (KSHV) ORF62 [13] and Epstein-Barr virus (EBV) BORF1 [14]. VP19Cs from alphaherpesviruses are larger than those in the beta and gamma herpesviruses and there is only 19–23% amino acid identity when comparing HSV-1 VP19C with HCMV and EBV orthologs, respectively. Nonetheless, they are functionally similar because they all form heterotrimers with their respective binding partners to stabilize the viral capsid.

Host restriction factors of the tripartite motif (TRIM) family of E3 ubiquitin ligases are well positioned to target such viral structures. Many TRIM proteins are interferon-stimulated genes and play important roles in intrinsic immunity [15]. For instance, TRIM25 destabilizes influenza A virus mRNAs and activates RIG-I signaling [16], whereas TRIM19 represses herpesviral transcription and traps capsids in the nucleus [17]. TRIM5α was identified as a selective, but potent, restriction factor for retroviruses for which it binds incoming capsids, accelerates their uncoating, and activates innate immune signaling [18,19]. However, its antiviral activity extends beyond retroviruses. TRIM5α also targets non-structural proteins of tick-borne flaviviruses and structural proteins of poxviruses and EBV [20–22]. These activities are all reliant on its E3 ubiquitin ligase activity, higher-order assembly, and the PRY–SPRY domain, yet the downstream consequences of this interaction vary across virus families. Engagement of EBV, retro-, or flaviviral proteins leads to their degradation, whereas interaction with the poxvirus capsid protein L3 (orthopoxvirus gene OPG097) stabilize L3 multimers, potentially disrupting L3 function. Therefore, TRIM5α represents a broad and versatile barrier to infection by several viruses.

Viruses have evolved diverse strategies to counteract TRIM5α. HIV-1 packages the cellular peptidyl prolyl isomerase Cyclophilin A (CypA) into virions to stabilize the capsid and prevent TRIM5α binding, an antagonism that can be neutralized by CypA inhibitors [21,23]. Poxviruses employ a dual strategy: the viral protein C6 (encoded by orthopoxvirus gene 29, OPG29) promotes TRIM5α degradation, while CypA interferes with TRIM5α-mediated L3 modification, enhanced L3 multimerization and innate signaling pathway activation [22]. Although TRIM5α restricts EBV by inducing its minor capsid protein degradation and interfering with virus gene expression, its role in other herpesviruses remains unclear. Three independent studies reported TRIM5 degradation during HSV-1 or HCMV infection [24–26], suggesting that TRIM5 might act against herpesviruses more broadly. CypA has also been detected in purified HSV-1 virions [27], but its role during infection and whether it operates in a TRIM5-dependent manner remain unknown. Thus, whether TRIM5 restricts HSV-1, which viral determinants mediate recognition, and if CypA influences this process are unresolved questions.

Here, we show that TRIM5α restricts HSV-1 infection and that this requires its E3 ubiquitin ligase activity, the PRY–SPRY domain, and the ability to oligomerize. In addition, we identify VP19C as the direct target of TRIM5α and show its expression can activate NF-kB signaling. HSV-1 counteracts TRIM5α-mediated restriction by promoting proteasome-dependent TRIM5α degradation. CypA also binds VP19C, but in contrast, acts as a proviral factor. Surprisingly, and in contrast to the situation with HIV-1 and poxviruses, the proviral activity of CypA is independent of TRIM5α. Nonetheless, the inhibition of CypA with cyclosporin A disrupts this interaction and suppresses viral replication. Finally, we show that TRIM5α and CypA interact strongly with orthologous capsid proteins from VZV, HCMV, EBV and KSHV, despite their differing sizes and low percentage amino acid identity, revealing conserved host–capsid interaction across the human herpesviruses. These findings identify TRIM5α and CypA as broad modulators of herpesvirus infection and highlight the host-capsid interface as a promising target for pan-herpesvirus antiviral development.

## Results

### TRIM5α restricts HSV-1 and is degraded during infection

**Fig 1:**
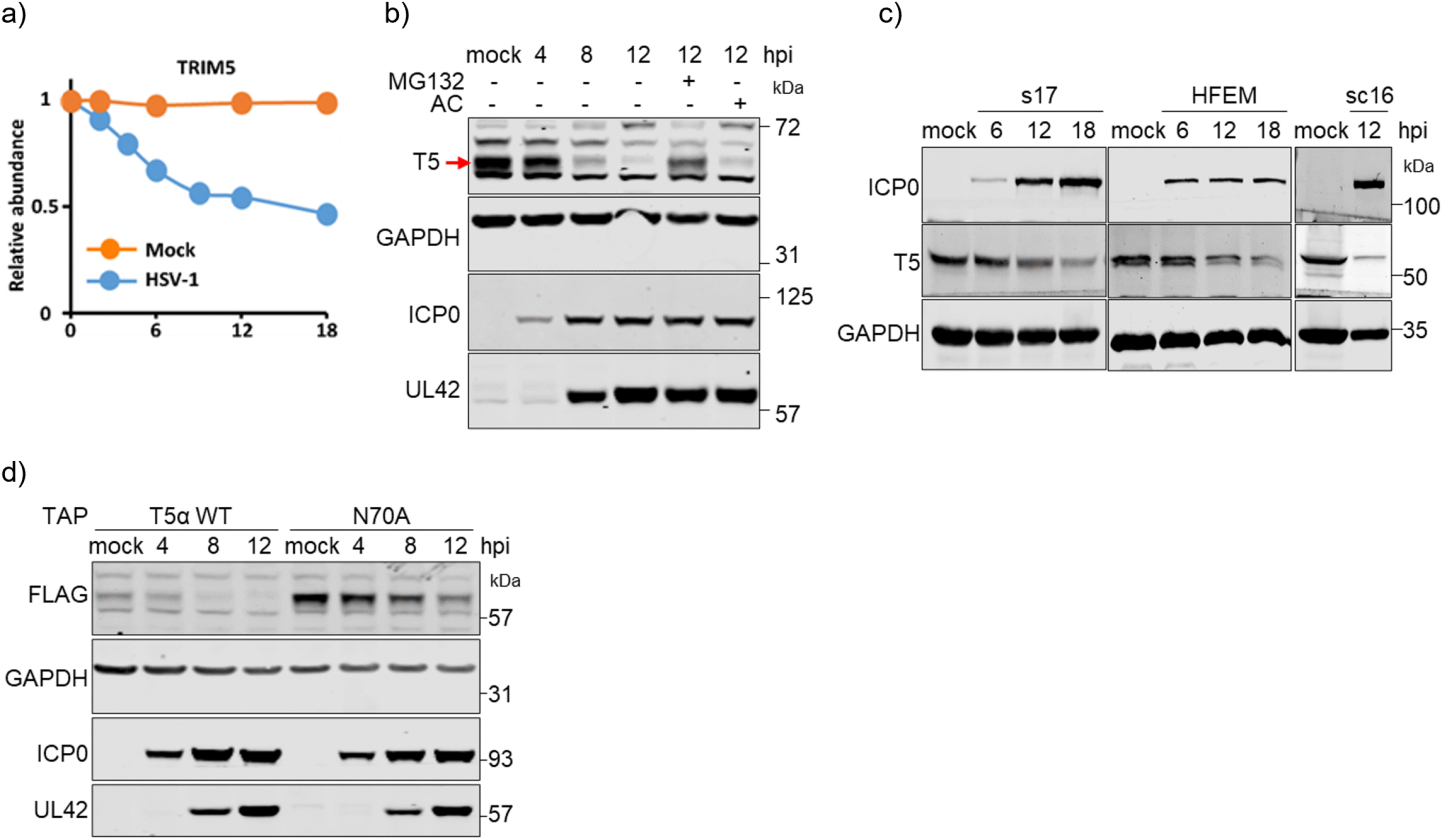
TRIM5 is degraded during infection. **a)** Temporal proteomic analysis of TRIM5 abundance during HSV-1 strain KOS infection in human keratinocytes. The graph is generated from data extracted from Soh et al., 2020 [24]. **b-d)** Immunoblotting of TRIM5α abundance during HSV-1 s17 infection in **b)** WT T-REx293 cells treated with (+) or without (-) MG132 or ammonium chloride (AC), **c)** U87-MG cells with HSV-1 s17, HFEM and sc16, **d)** TRIM5^-/-^ T-REx293 cells inducibly expressing TAP-tagged WT TRIM5α and N70A at 4/6, 8 and 12/18 hours post infection (hpi) at 5 pfu/cell. Data shown are representative of three independent experiments.

Mass spectrometry of HSV-1 strain KOS-infected human keratinocytes showed that TRIM5 was reduced in abundance as infection progressed [24] (Fig 1a). This viral-induced degradation was also observed in HeLa cells [26]. Immunoblotting showed TRIM5 degradation in T-REx293 cells after infection with HSV-1 strain 17 (s17) (Fig 1b), and in human glioblastoma cells after infection with HSV-1 strains s17, HFEM and sc16 (Fig 1c). The degradation was proteasome-dependent because the addition of MG132, but not the lysosomal inhibitor ammonium chloride, prevented TRIM5 downregulation (Fig 1b). HSV-1-induced TRIM5 degradation did not require autoubiquitylation of TRIM5 because a mutant TRIM5, N70A, which lacks this activity, was still reduced after HSV-1 infection (Fig 1d).

**Fig 2:**
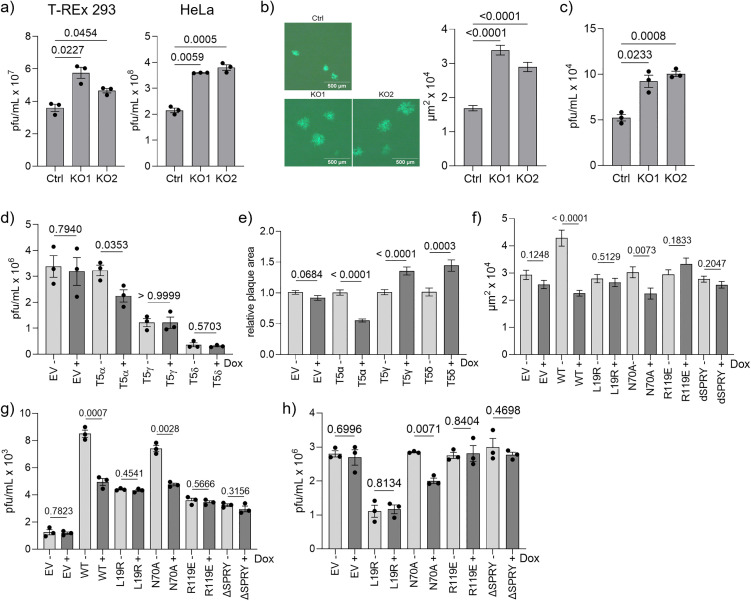
TRIM5α restricts HSV-1. **a)** HSV-1 s17 infectious virus titres following infection at 5 pfu/cell in WT and TRIM5^-/-^ T-REx293 and HeLa at 10 hpi. n = 3/condition. Ctrl = control. KO1 and KO2 = TRIM5 knockout cell lines 1 and 2 in indicated cell type. **b)** Plaque image (left) and plaque area quantification (right) of GFP-VP26 HSV-1 s17 following infection to give well separated plaques in WT and TRIM5^-/-^ HeLa cells at 24 hpi. **c)** HSV-1 s17 infectious virus titres following infection at 0.01 pfu/cell in WT and TRIM5^-/-^ T-REx293 at 24 hpi. n = 3/condition. **d**) HSV-1 s17 infectious virus titres following infection at 5 pfu/cell in TRIM5^-/-^ T-REx293 inducibly expressing TAP-tagged TRIM5α, -γ and –δ at 10 hpi. n = 3/condition. **e)** Relative plaque area quantification of GFP-VP26 HSV-1 s17 following infection in WT T-REx293 cells inducibly expressing TAP-tagged TRIM5α, -γ and –δ at 24 hpi. n > 38/condition. **f)** Plaque area quantification following infection of GFP-VP26 HSV-1 s17 in TRIM5^-/-^ T-REx293 cells inducibly expressing TAP-tagged EV, WT T5α, L19R, N70A, R119E and ∆SPRY at 24 hpi. n > 34/condition. **g)** HSV-1 s17 infectious virus titres following infection at 0.001 pfu/cell in cells described in **f)** at 24 hpi. n = 3/condition. **h)** HSV-1 s17 infectious virus titres following infection at 5 pfu/cell in cells described in **f)** at 10 hpi. n = 3/condition. Data shown are representative of three independent experiments. Data from **a-c)** were analyzed using one-way Welch’s analysis of variance (ANOVA) test. Data from **d-h)** were analyzed using two-tailed unpaired Student’s *t*-tes*t*. Analyses were performed on GraphPad Prism. Data are mean ± s.e.m.

A hypothesis to explain TRIM5 degradation was that TRIM5 has anti-HSV-1 activity and, therefore, HSV-1 has evolved a strategy to remove it. Indeed, the infectious virus yield following high multiplicity of infection (MOI) in T-REx293 cells or HeLa cells (Fig 2a) that were engineered to lack all TRIM5 isoforms [22] were enhanced compared to parental cells. Similarly, the plaque size of GFP-tagged HSV-1 in HeLa cells (Fig 2b), and the infectious virus yields after low MOI in TRIM5^-/-^ T-REx293 (Fig 2c) cells were enhanced compared to their respective parental control cell lines, showing that TRIM5 restricts HSV-1 replication and/or spread.

Alternative splicing produces several TRIM5 mRNAs encoding isoforms with different C termini, some of which are antiviral, whilst others act as their dominant negative regulators [22,28]. To identify which isoform(s) exhibit(s) anti-HSV-1 activity and to ensure the phenotypes observed in the TRIM5^-/-^ cell lines are not due to off-target effects of CRISPR-Cas9, three TRIM5 isoforms, TRIM5α, -γ and -δ, were individually expressed in an inducible Tet-on system in T-REx293 TRIM5^-/-^ cells [22]. The expression of TRIM5α reduced virus titres after high MOI, whereas TRIM5γ and TRIM5δ expression had no effect (Fig 2d), pinpointing TRIM5α as the HSV-1 restriction factor. Moreover, whereas over-expression of TRIM5α in WT T-REx293 cells reduced HSV-1 plaque size, TRIM5γ and -δ enhanced virus spread, suggesting these isoforms exert a dominant negative effect on endogenous TRIM5α-mediated restriction (Fig 2e).

To further dissect the antiviral activity of TRIM5α, infectious virus yields after high and low MOI, and plaque sizes in T-REx293 TRIM5^-/-^ cell lines that inducibly express TRIM5α mutants that lack enzymatic activity, oligomerization or functional domains were assessed (Fig 2f-2h). L19R is defective in E3 ubiquitin ligase activity [29] and therefore cannot monoubiquitylate or synthesize anchored or unanchored polyubiquitin chains. N70A cannot monoubiquitylate and form anchored polyubiquitin chains, but can still activate innate immune signaling pathways such as NF-κB and AP1 [29]. R119E is impaired in TRIM5α oligomerization [30] and ΔSPRY lacks the C-terminal PRY-SPRY domain, which is needed to recognize viral substrates from retroviruses [31], flaviviruses [21] and poxviruses [22]. The inducible expression of N70A reduced infectious virus yields and plaque size in TRIM5^-/-^ cells after high and low MOI, whereas the expression of L19R, R119E or ΔSPRY was not inhibitory. This suggests that the antiviral activity of TRIM5α involves unanchored polyubiquitin chain synthesis, oligomerization and its C-terminal domain, possibly to recognize and bind viral substrate(s).

### TRIM5α binds to the HSV-1 capsid protein VP19C

**Fig 3:**
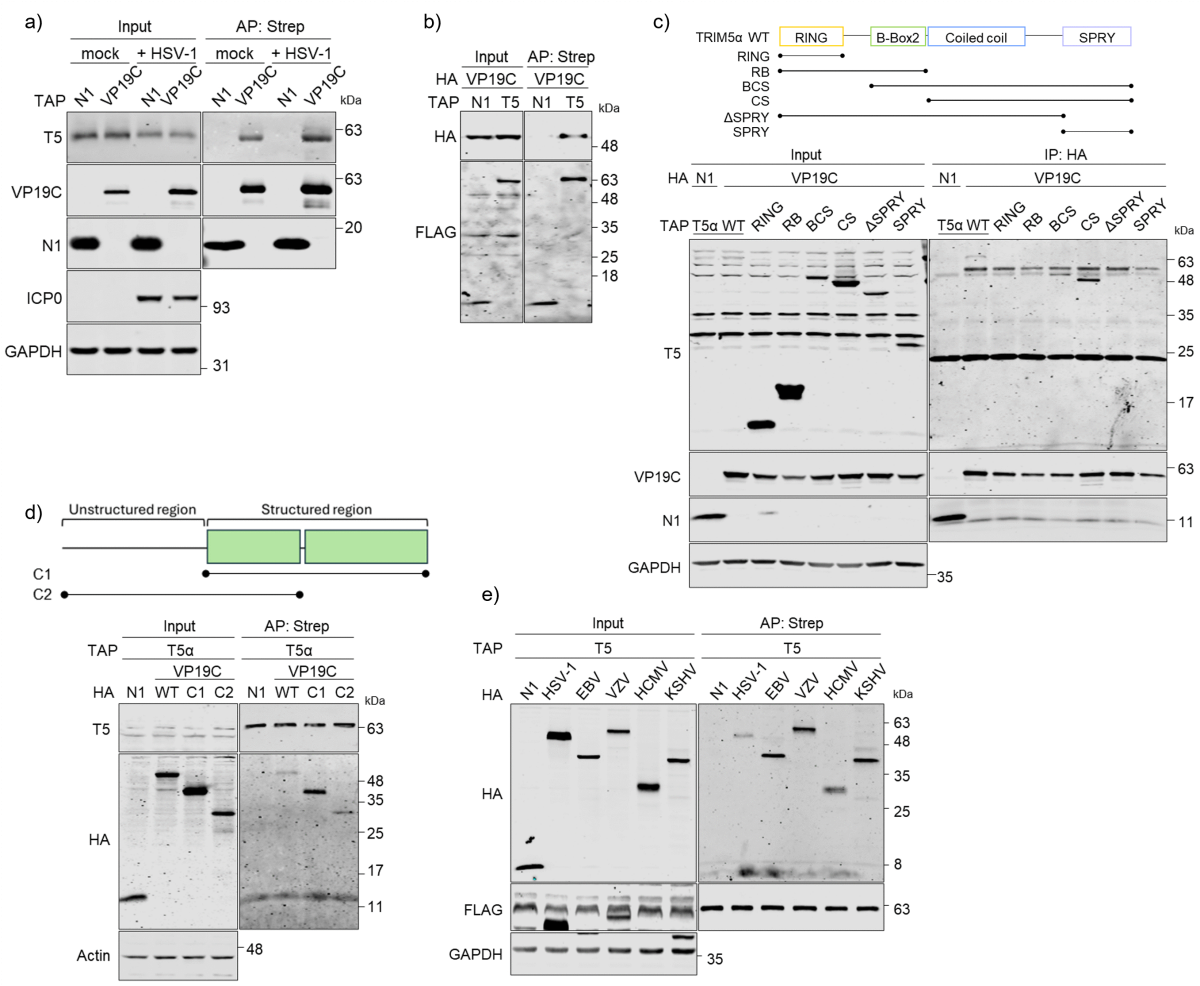
TRIM5α binds to the HSV-1 capsid protein VP19C. **a)** Endogenous TRIM5α co-precipitates with VP19C with and without infection. T-REx293 cells were transfected to express TAP-tagged VP19C and N1 (negative control) for 24 h before either harvest for mock-infected samples, or infection with HSV-1 s17 at 3 pfu/cell for 16 h for infected samples. Affinity purification was carried out using TAP-tagged proteins as baits followed by immunoblotting. **b)** TRIM5α and VP19C interact directly. TAP-tagged N1 (negative control) and TRIM5α were co-expressed with HA-tagged VP19C in the wheat germ transcription and translation system followed by affinity purification in the presence of benzonase and immunoblotting. **c)** TRIM5α interacts with VP19C via its C-terminal domains. TAP-tagged WT and domain deletion mutants of TRIM5α (schematic of TRIM5α protein domains and deletion mutants shown above) were co-expressed with HA-tagged N1 (negative control) or VP19C in TRIM5^-/-^ T-REx293 cells for 24 h before harvesting for affinity purification and immunoblotting. RB = RING and B-Box2; BCS = B-Box2, coiled-coil and SPRY; CS = coiled-coil and SPRY; ΔSPRY = TRIM5α lacking SPRY domain. **d)** VP19C interacts with TRIM5α via its C-terminal domains. TAP-tagged WT TRIM5α was co-expressed with HA-tagged N1 (negative control), WT or domain deletions of VP19C (schematic of VP19C protein domains and deletion mutants shown above) in TRIM5^-/-^ T-REx293 cells for 24 h before harvesting for affinity purification and immunoblotting. **e)** TRIM5α interacts with VP19C orthologues in EBV, VZV, HCMV and KSHV. TAP-tagged TRIM5α was co-expressed with HA-tagged N1 (negative control), VP19C or its orthologs in TRIM5^-/-^ T-REx293 cells for 24 h before harvesting for affinity purification and immunoblotting. Data shown in **a)**, **b)** and **e)** are representative of two independent experiments and data shown in **c)** and **d)** are representative of three independent experiments.

TRIM5α binds to structural proteins from retroviruses [31], poxviruses [22] and EBV [20] and non-structural proteins from flaviviruses [21]. For EBV, the TRIM5α target is structural protein, BORF1, which interacts with EBV protein BDLF1. The HSV-1 orthologs, VP19C and VP23 also interact to form heterotrimers and stabilize HSV-1 capsids [32]. Therefore, the possible interaction of TRIM5α and VP19C was investigated and the vaccinia virus (VACV) protein N1 (orthopoxvirus gene 35), an early protein that inhibits NF-κB [33], was used as the negative control. Due to the lack of available antibody, VP19C was expressed with an epitope-tag. Endogenous TRIM5α and VP19C co-precipitated in the presence and absence of HSV-1 (Fig 3a), indicating this interaction occurs during HSV-1 infection and does not require other viral proteins. The expression of TRIM5α and VP19C in the wheat germ transcription and translation system followed by affinity purification in the presence of nucleases showed that this interaction does not require other mammalian or viral proteins, and therefore, is likely to be direct (Fig 3b).

To map the region of TRIM5α needed for interaction, TRIM5α mutants that lack one or more domains (Fig 3c) were co-expressed with HA-tagged VP19C and tested for co-precipitation. VP19C-TRIM5α interaction does not require the N-terminal RING and B-Box2 domains of TRIM5α (Fig 3c). However, the PRY-SPRY domain alone was insufficient to bind to VP19C, probably due to the need for TRIM5α multimerization. Next, the region of VP19C needed for TRIM5α interaction was investigated. VP19C has an unstructured N-terminal region of 66 amino acids, containing an NLS, and is followed by a structured C-terminal region (Fig 3d) [6]. Co-expression and co-precipitation with TRIM5α showed that the N- and C-terminal regions are dispensable for interaction with TRIM5α (Fig 3d).

VP19C and BORF1 orthologs exist in other herpesviruses. Amino acid sequence alignment of VP19C and BORF1 with their orthologs in other herpesvirus such as VZV ORF20 [11], HCMV UL46 [12] and KSHV ORF62 [13] revealed low (19–23%) overall amino acid sequence identity and that the alphaherpesvirus proteins are larger than their beta and gammaherpesvirus counterparts. Nonetheless, these other herpesvirus proteins all showed stronger co-precipitation with TRIM5α (Fig 3e), suggesting the broad scope of TRIM5α-mediated herpesvirus restriction may depend on conserved protein structure rather than amino acid sequences.

### TRIM5α affects VP19C-VP23 interaction and localization

**Fig 4:**
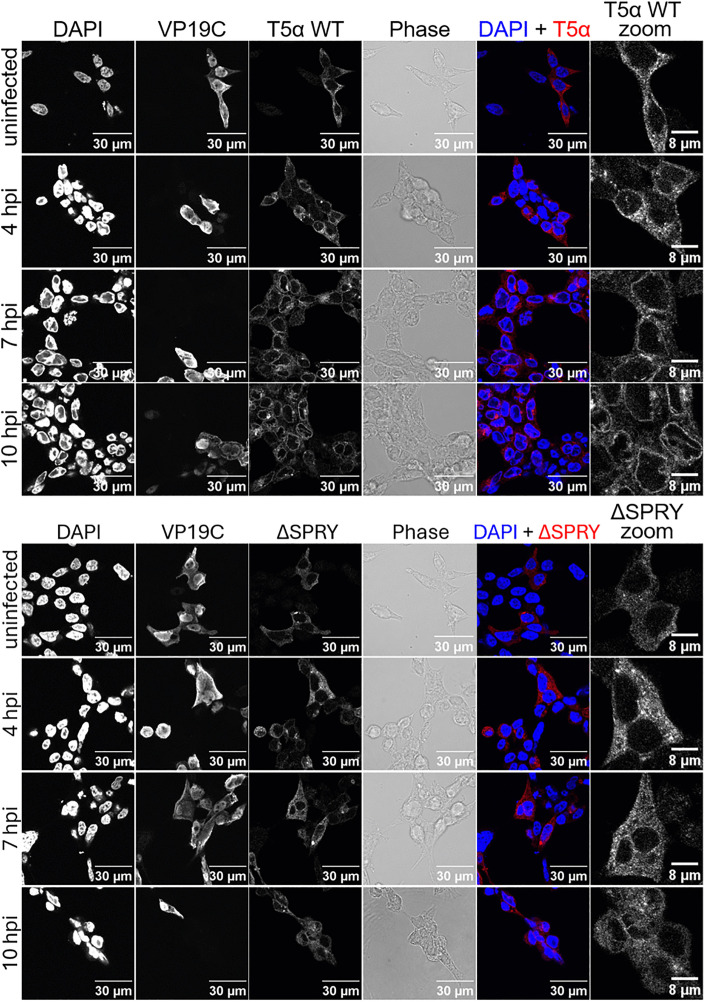
TRIM5α localization during HSV-1 s17 infection. TRIM5^-/-^ T-REx293 cells inducibly expressing TAP-tagged TRIM5α and transfected with a plasmid encoding HA-tagged VP19C were infected with HSV-1 s17 at 5 pfu/cell for 0, 4, 7 and 10 h**.** Cells were stained with DAPI (blue) and antibodies against FLAG (TRIM5α, red) and HA (VP19C). Images shown are representative of at least five images/condition and representative of three independent experiments.

TRIM5α might mediate its anti-HSV-1 activity in several ways via its interaction with VP19C. TRIM5α could recognize incoming virions after uncoating exposes nucleocapsids in the cytoplasm. Alternatively, TRIM5α could interfere with VP19C functions either during its nuclear entry with other capsid proteins, VP23 or VP5, or during nuclear exit as an assembled capsid. To start to address these possibilities, the localisation of TRIM5α was examined before and after HSV-1 infection and was found to re-distribute to the nuclear periphery, possibly to the nuclear membrane late during infection (Fig 4). Given this localization, the latter hypotheses seemed more probable. VP19C was transiently transfected into TRIM5^-/-^ cells expressing tagged TRIM5α to determine if TRIM5α and VP19C co-localized, however the high level of VP19C expression resulted in its aberrant localization [9] and prevented conclusions being reached. Nevertheless, we did note that the redistribution of TRIM5α to the nuclear membrane was unaffected by VP19C over-expression.

**Fig 5:**
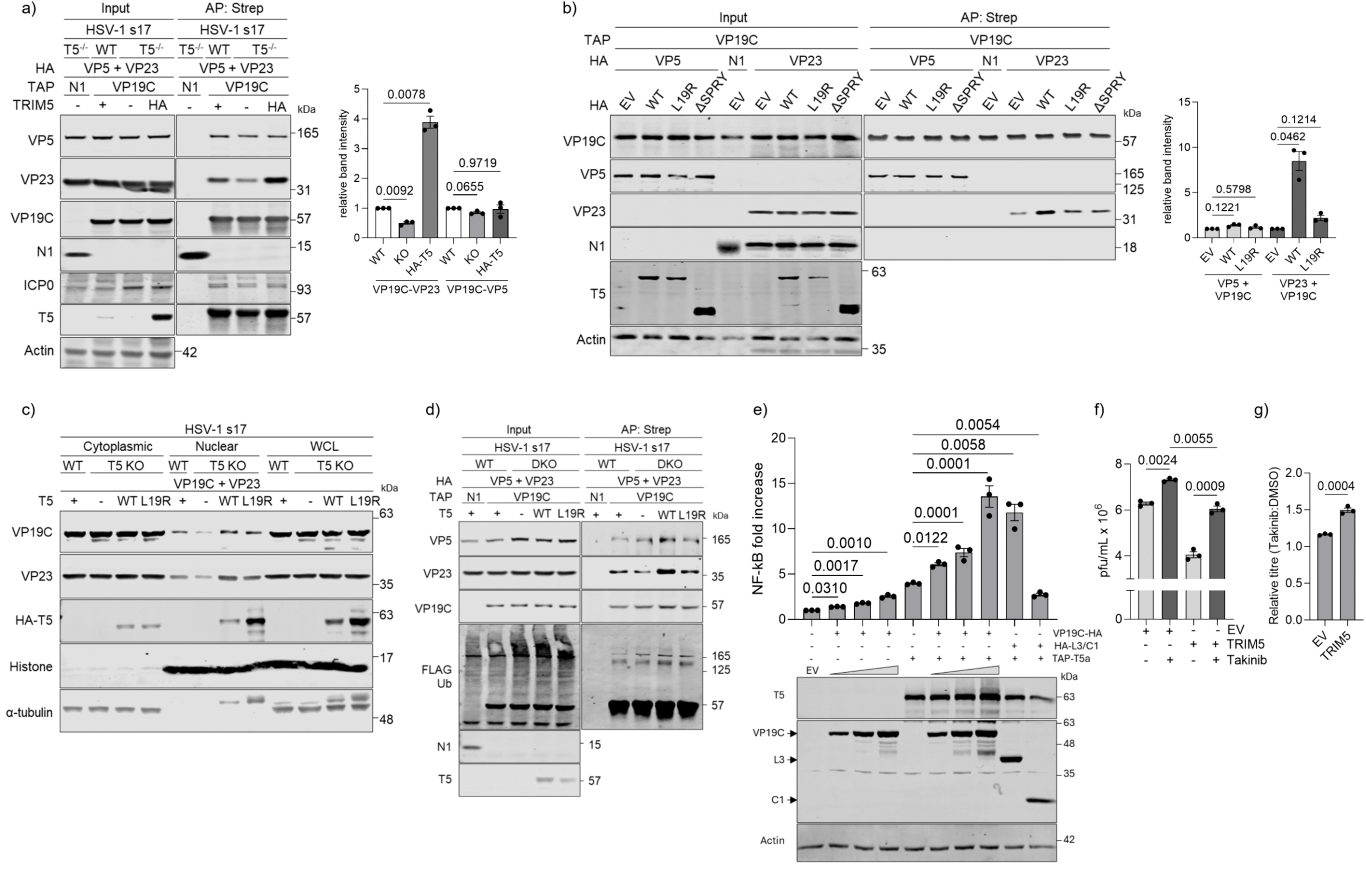
Consequences of TRIM5α and VP19C interaction. **a)** VP19C-VP23 interaction is enhanced in the presence of TRIM5α. TAP-tagged N1 (negative control) or VP19C were co-expressed with HA-tagged VP23 in WT, TRIM5^-/-^ or TRIM5^-/-^ T-REx293 cells expressing HA-tagged TRIM5α. Transfected cells were infected with HSV-1 s17 for 16 h before harvesting for affinity purification and immunoblotting. Band intensity quantification (right) is calculated as a ratio of VP23/VP5:VP19C band intensity of AP fractions from three independent experiments. **b)** TRIM5α-mediated enhancement of VP19C-VP23 interaction requires its E3 ubiquitin ligase activity. TAP-tagged VP19C and HA-tagged N1 (negative control), VP5 or VP23 were co-expressed with HA-tagged EV, WT TRIM5α, L19R or ∆SPRY for 24 h before harvesting for affinity purification and immunoblotting. Bands detected (~18 kDa) in VP23 expression conditions of the N1 input panel are degraded products of VP23. Band intensity quantification from three independent experiments is shown on the right. **c)** TRIM5α increases the abundance of VP19C and VP23 in the nuclear fraction. HA-tagged VP19C was co-expressed with TAP-tagged VP23 in TRIM5^+/+^, TRIM5^-/-^ or TRIM5^-/-^ T-REx293 cells expressing HA-tagged WT TRIM5α or L19R mutant. Transfected cells were infected with HSV-1 s17 for 16 h before harvesting for fractionation and immunoblotting. **d)** Does TRIM5α ubiquitylate VP19C during infection? TAP-tagged N1 (negative control) or VP19C were co-expressed with HA-tagged VP5, VP23 and FLAG-tagged ubiquitin in TRIM5^+/+^, TRIM5^-/-^ or TRIM5^-/-^ T-REx293 expressing HA-tagged WT TRIM5α or L19R. Cells were infected with HSV-1 s17 for 16 h before harvesting for affinity purification and immunoblotting. **e)** TRIM5α and VP19C activate NF-κB synergistically. TRIM5^-/-^ T-REx293 cells were co-transfected with 100 ng NF-κB-luciferase and 10 ng *Renilla* luciferase alongside either EV, VP19C, L3 (positive control) or WT TRIM5α, individually, or WT TRIM5α with VP19C, L3 or C1 (negative control). Cells were harvested 24 h after transfection, firefly luciferase activity was measured and normalized to *Renilla* luciferase. Fold induction is relative to EV. n = 3/condition. **f)** Pharmacological inhibition of TAK1 reduces TRIM5α-mediated HSV-1 restriction. TRIM5^-/-^ T-Rex293 cells expressing EV or TRIM5α were pretreated with DMSO or 3 µM Takinib for 12 h and then infected with HSV-1 s17 at 0.01 pfu/cell for 24 h in the presence or absence of Takinib. The yield of infectious virus was determined by plaque assay. **g)** Takinib promotes higher infectious virus titres in the presence of TRIM5α. Relative virus titres (Takinib:DMSO) in TRIM5^-/-^ T-Rex293 cells expressing EV or TRIM5α, pretreated with DMSO or 3 µM Takinib for 12 h and infected with HSV-1 s17 at 0.01 pfu/cell for 24 h in the presence or absence of Takinib. Data shown in **a-c)** and **e)** are representative of three independent experiments and data shown in **d)** and **f)** are from two independent experiments. Data from **a)**, **b)**, **e-g)** were analyzed using one-way Welch’s analysis of variance (ANOVA) test. Analyses were performed on GraphPad Prism. Data are mean ± s.e.m.

To explore the consequence of VP19C-TRIM5α binding, VP19C interactions with VP23 and VP5 in the presence or absence of TRIM5α was assessed. Due to lack of available antibodies against the HSV-1 capsid proteins, epitope-tagged proteins were co-expressed during HSV-1 infection. The interaction between VP19C and VP23 was enhanced in the presence of endogenous and HA-tagged TRIM5α, whereas VP19C-VP5 was unaffected, showing targeted restriction on VP19C-VP23 interaction (Fig 5a). Furthermore, this TRIM5α-mediated stabilization of interaction requires the E3 ubiquitin ligase and the PRY-SPRY domain because the mutants, L19R and ΔSPRY, were unable to affect VP19C-VP23 co-precipitation (Fig 5b).

To understand whether the enhanced interaction affects VP19C and VP23 subcellular distribution, fractionation of HSV-1-infected cells co-expressing VP19C and VP23 was undertaken. Whilst the abundance of VP19C and VP23 in the cytoplasmic fraction were unaffected by the presence or absence of TRIM5α, there was notably more VP19C and VP23 in the nuclear fractions when TRIM5α was present, either at endogenous levels or when expressed ectopically (Fig 5c). Moreover, this altered distribution is mediated by the E3 ubiquitin ligase activity of TRIM5α, because expression of the catalytically impaired mutant, L19R, resulted in reduced abundance of nuclear VP19C and VP23 compared to the WT TRIM5α.

Given that both the enhanced VP19C-VP23 interaction and nuclear localization depend on the E3 ubiquitin ligase activity of TRIM5α, an enzymatic activity critical to its restriction against other viruses, ubiquitylation of VP19C was investigated. However, under the conditions tested, ubiquitylation was not observed (Fig 5d).

### VP19C activates NF-κB

For retroviruses and poxviruses, there are at least two mechanisms of TRIM5α-mediated restriction: direct inhibition of virus replication and activation of innate immune signaling pathways [19,22]. Given that the partially impaired E3 ubiquitin ligase mutant, N70A, which induces greater activation of NF-κB than even WT TRIM5α [22], still exhibits anti-viral activity, it is possible that its restriction occurs via NF-κB activation (Fig 2f-2h). However, because HSV-1 encodes multiple NF-κB inhibitors, assessing NF-κB activation during infection is likely to mask the specific contribution of VP19C. Therefore, NF-κB reporter gene assays were undertaken in cell lines lacking endogenous TRIM5α outwith infection to address whether VP19C induces innate signaling activation (Fig 5e). Ectopic expression of VP19C induced NF-κB activation in a dose-dependent manner and activation was enhanced when VP19C and TRIM5α were co-expressed, indicating that VP19C can be recognized by innate immune sensor(s) independent of TRIM5α, but together they potentiate this response. TRIM5α synthesizes unanchored polyubiquitin chains and activates NF-κB at the level of TAK1 during retroviral infection [19]. Pharmacological inhibition of TAK1 promoted HSV-1 replication and/or spread (Fig 5f) but less so in the absence of TRIM5α (Fig 5g), indicating that TRIM5α restricts HSV-1 by activating NF-κB signalling through TAK1. However, TRIM5 expressing cells still yielded lower virus titres compared to TRIM5^-/-^ cells, despite TAK1 inhibitor treatment (Fig 5f), showing that there is at least two mechanisms of TRIM5α-mediated restriction, possibly by regulating VP19C-VP23 interaction.

### CypA is proviral and promotes HSV-1 replication

Cyclophilin A (CypA) is a peptidyl-prolyl isomerase that catalyzes the cis-trans isomerization of the peptide bond preceding proline residues on its substrates [34]. Its interaction with its inhibitor, cyclosporine A (CsA) results in the inactivation of T-cells [35]. CypA enhances replication of several viruses including flaviviruses, MERS-CoV, SARS-CoV-2, HIV-1 and VACV [22,36–39]. For HIV-1 and VACV, CypA is packaged into virions and is shown to antagonize the antiviral activity of TRIM5α in both cases [23,40,41]. CypA is also incorporated into influenza A virus virions, although it restricts replication rather than aids infection [42,43]. Mass spectrometry of purified HSV-1 virions also detected CypA [27], but whether this is advantageous to the virus is unknown.

**Fig 6:**
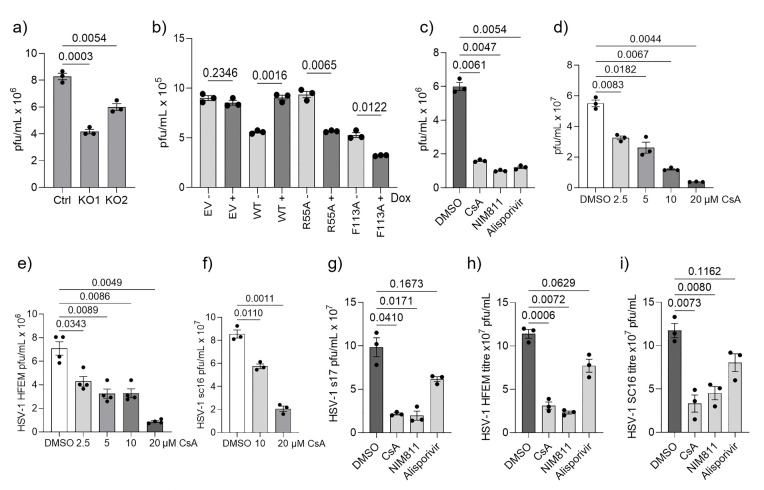
CypA is proviral and CsA is antiviral. **a-d)** HSV-1 s17 infectious virus titres following infection at 5 pfu/cell in **a)** WT and CypA^-/-^ T-REx293 cells, **b)** CypA^-/-^ T-REx293 cells inducibly expressing TAP-tagged EV, WT or catalytically defective CypA mutants, R55A and F113A, **c)** WT T-REx293 treated with DMSO or CsA, NIM811 and alisporivir at 5 µM and **d)** U87-MG cells treated with DMSO or CsA at 2.5, 5, 10 and 20 µM at 10 hpi. n = 3/condition. **e)** HSV-1 HFEM and **f)** HSV-1 sc16 infectious virus titres following infection at 5 pfu/cell in U87-MG cells treated with DMSO or CsA at 2.5, 5, 10 and 20 µM at 10 hpi. n = 3/condition. **g)** HSV-1 s17, **h)** HSV-1 HFEM and **i)** HSV-1 SC16 infectious virus titres following infection at 5 pfu/cell in U87-MG cells treated with DMSO, CsA, NIM811 or alisporivir at 5 µM at 10 hpi. n = 3/condition. Data shown in **a)**, **b)**, **d)**, and **g-i)** are representative of three independent experiments and data shown in **c), e)** and **f)** are from two independent experiments. Data from **a)**, **c)** and **d-i)** were analyzed using one-way Welch’s analysis of variance (ANOVA) test. Data from **b)** was analyzed using two-tailed unpaired Student’s *t*-test. Analyses were performed on GraphPad Prism. Data are mean ± s.e.m.

The yield of infectious virus in CypA^-/-^ T-REx293 cells following HSV-1 infection at 5 pfu/cell was reduced compared to the WT control, showing that CypA is proviral and promotes HSV-1 replication (Fig 6a). This requires the enzymatic activity of CypA because the inducible expression of catalytically inactive mutants, R55A and F113A in CypA^-/-^ cells, restricted, rather than rescued, virus replication (Fig 6b). Conversely, inhibition of CypA during HSV-1 infection by its inhibitor, CsA, and its non-immunosuppressive derivatives, NIM811 and alisporivir, reduced infectious virus yields compared to the DMSO-treated control, showing that these drugs restrict HSV-1 (Fig 6c). Cell viability was unaltered at these drug concentrations. The anti-viral activity of CsA and its derivatives was also observed in HSV-1 s17, HFEM and sc16-infected glioblastoma cells (Fig 6d-6i), demonstrating the potential to use CypA inhibitors as antivirals in cell types that support HSV-1 latent and lytic infections.

**Fig 7:**
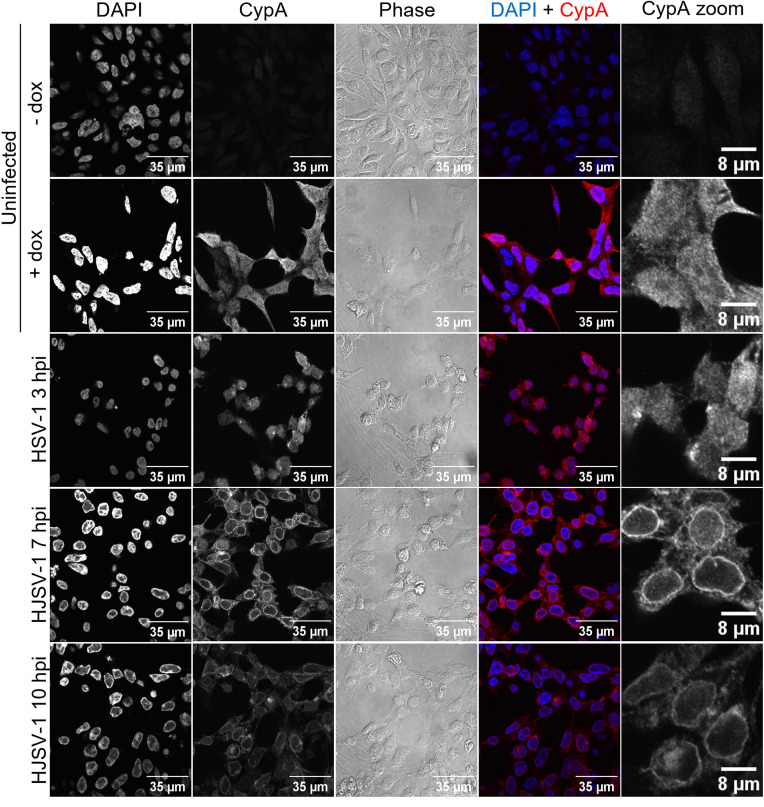
CypA localization during HSV-1 s17 infection. CypA^-/-^ T-REx293 cells inducibly expressing TAP-tagged CypA were infected with HSV-1 s17 at 5 pfu/cell for 0, 3, 7 and 10 h. Cells were stained with DAPI (blue) and antibodies against FLAG (red). Images shown are representative of at least five images/condition. Data shown is representative of two independent experiments.

The localization of CypA during HSV-1 infection was investigated to explore its proviral mechanism. CypA^-/-^ T-REx293 cells were induced to express TAP-tagged CypA prior to infection with HSV-1 s17 at 5 pfu/cell (Fig 7). Whereas CypA was distributed throughout uninfected cells, there was a clear re-localization to surround the nucleus by 7 and 10 h, suggesting its proviral activity might be deployed late during infection, possibly aiding viral protein nuclear import or capsid export.

### CypA binds to VP19C

**Fig 8:**
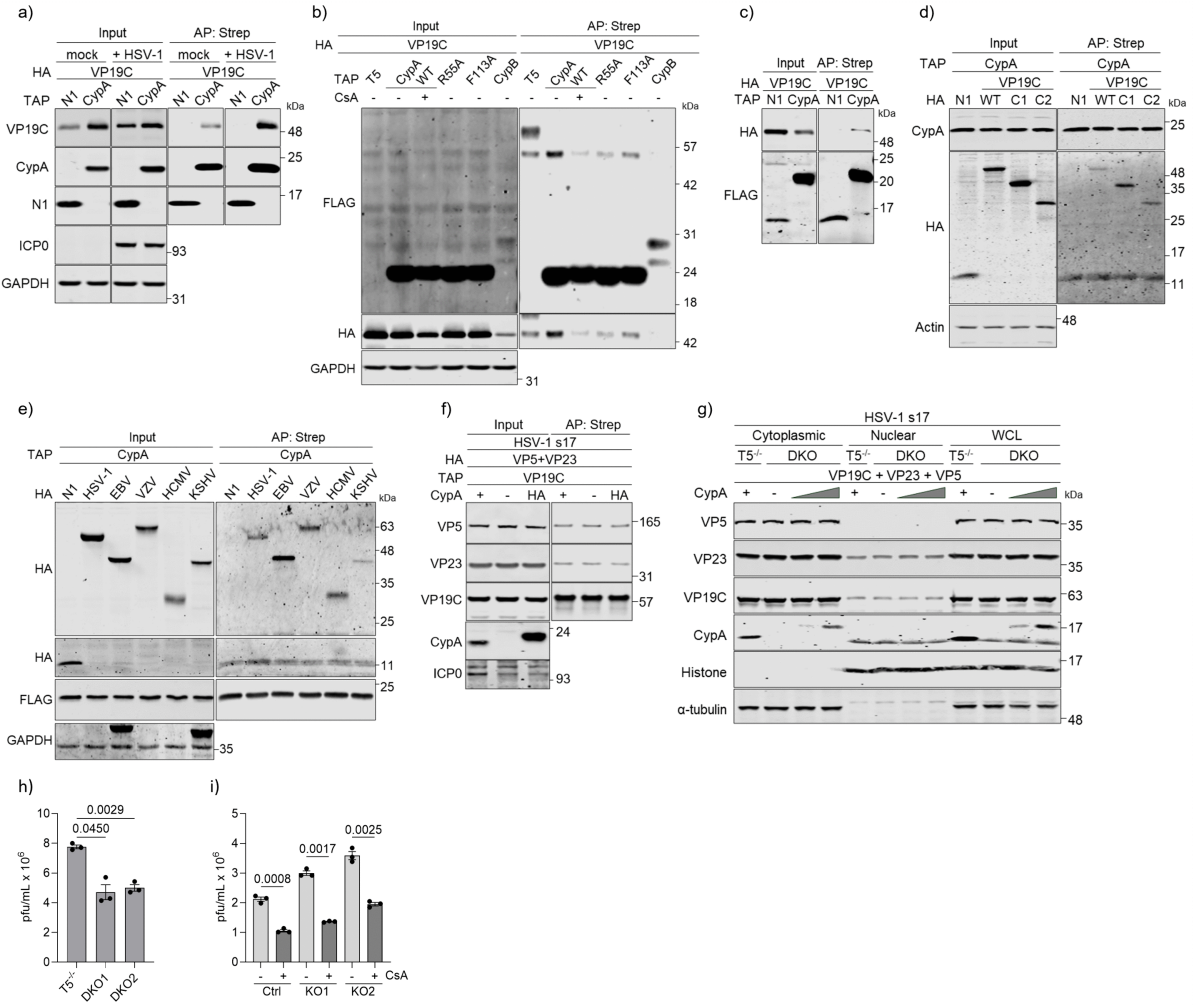
CypA binds to VP19C. **a-b)** TAP-tagged CypA co-precipitates with VP19C **a)** with and **b)** without infection. T-REx293 cells were transfected to express TAP-tagged N1 (negative control), WT CypA or catalytically defective CypA mutants, R55A and F113A, for 24 h before harvest **b)** for mock-infected samples or **a)** infection with HSV-1 s17 at 3 pfu/cell for 16 h for infected samples. Affinity purification was carried out using TAP-tagged proteins as baits followed by immunoblotting. **c)** CypA and VP19C interact directly. TAP-tagged N1 (negative control) and CypA were co-expressed with HA-tagged VP19C in the wheat germ transcription and translation system followed by affinity purification in the presence of nucleases and immunoblotting. **d)** VP19C interacts with CypA via its C-terminal domains. TAP-tagged CypA was co-expressed with HA-tagged N1 (negative control), WT or domain deletions of VP19C in CypA^-/-^ T-REx293 cells for 24 h before harvesting for affinity purification and immunoblotting. **e)** CypA interacts with VP19C orthologs in EBV, VZV, HCMV and KSHV. TAP-tagged CypA was co-expressed with HA-tagged N1 (negative control), VP19C or its orthologs in TRIM5^-/-^ T-REx293 cells for 24 h before harvesting for affinity purification and immunoblotting. **f)** CypA does not influence VP19C interaction with its viral binding partners. TAP-tagged VP19C was co-expressed with HA-tagged VP23 and VP5 in CypA^-/-^ or CypA^-/-^ T-REx293 cells expressing HA-tagged CypA. Transfected cells were infected with HSV-1 s17 for 16 h before harvesting for affinity purification and immunoblotting. **g)** CypA does not influence the subcellular distribution of VP19C or its viral binding partners. TAP-tagged VP19C was co-expressed with HA-tagged VP5 and VP23 in CypA^+/+^, CypA^-/-^ or CypA^-/-^ T-REx293 cells expressing increasing concentrations of HA-tagged CypA. Transfected cells were infected with HSV-1 s17 for 16 h before harvesting for fractionation and immunoblotting. **h)** HSV-1 s17 infectious virus titres following infection at 5 pfu/cell in TRIM5^-/-^ and TRIM5^-/-^ CypA^-/-^ (double knockout, DKO1 and DKO2) T-REx293 cells at 10 hpi. n = 3/condition. **i)** HSV-1 s17 infectious virus titres following infection at 5 pfu/cell in WT or TRIM5^-/-^ T-REx293 treated with 5 µM CsA at 10 hpi. n = 3/condition. Data shown in **b)**, **e)**, **f)** and **g)** are representative of three independent experiments and data shown in **a)**, **c)**, **d)**, **h)** and **i)** are from two independent experiments. Data from **h)** was analyzed using one-way Welch’s analysis of variance (ANOVA) test. Data from **b)** was analyzed using two-tailed unpaired Student’s *t*-test. Analyses were performed on GraphPad Prism. Data are mean ± s.e.m.

Thus far, CypA has shown a similar virion association and proviral role during retro-, pox- and herpesvirus infections. Therefore, by analogy with retro- and poxviruses, we hypothesized that CypA may also bind to the same herpesvirus capsid protein as TRIM5α. CypA was co-expressed with VP19C and co-precipitation was observed in both infected and uninfected cells (Fig 8a), showing the interaction does not require other viral proteins. Notably, the abundance of co-precipitated VP19C was reduced in cells treated with CsA (Fig 8b), suggesting the mechanism of CsA antiviral activity might occur via disrupting CypA-VP19C interaction. Additionally, VP19C co-precipitated with the catalytically defective mutants, albeit at lower levels, suggesting that these mutants exert an antiviral effect by engaging the viral substrate in a manner that interferes with viral replication. Furthermore, the co-expression of CypA and VP19C in the wheat germ transcription and translation system followed by affinity purification in the presence of nucleases, indicates this interaction does not require other mammalian or viral proteins, and is likely to be direct (Fig 8c). As for the TRIM5α-VP19C interaction, the N- and C-terminal regions of VP19C are dispensable for CypA-VP19C interaction (Fig 8d).

To investigate the extent of CypA binding to other human herpesvirus capsid proteins, ORF20 (VZV), UL46 (HCMV), ORF62 (KSHV) and BORF1 (EBV) were co-expressed with CypA. Affinity purification demonstrated CypA interaction with capsid proteins from members of the *alpha-*, *beta-* and *gammaherpesvirinae* (Fig 8e). Therefore, CypA might influence infection by multiple human herpesviruses, although whether it is proviral or antiviral remains to be elucidated.

Next, the consequence of VP19C-CypA interaction was addressed. VP5 and VP23 were co-expressed with VP19C during HSV-1 infection in the presence and absence of endogenous or HA-tagged CypA to assess strength of co-precipitation (Fig 8f) and changes in subcellular localization (Fig 8g). However, neither binding nor nuclear/cytoplasmic abundance of VP23 or VP5 was affected by CypA expression.

### CypA is proviral but TRIM5-independent

Similar to their roles and interactions with the same viral proteins during retro- and poxvirus infections, here, we show that TRIM5α and CypA also exhibit antiviral and proviral activities during HSV-1 infection, respectively, and bind to the same HSV-1 capsid protein, VP19C (Figs 3a, 8a). Given the TRIM5-dependent proviral activity of CypA in retroviruses and poxviruses, we investigated if the pro-HSV-1 activity of CypA was also TRIM5α dependent. Infectious virus titres in TRIM5^-/-^ and TRIM5^-/-^ CypA^-/-^ T-REx293 cells following HSV-1 s17 infection was quantified (Fig 8h). Lack of CypA expression reduced virus titres regardless of the presence or absence of TRIM5, showing an independency of these cellular factors in their respective roles in HSV-1 infection of these cells. Furthermore, the antiviral activity of CsA is also TRIM5-independent because its effect was observed in both WT and TRIM5^-/-^ T-REx293 cells (Fig 8i).

## Discussion

This study broadens the antiviral scope of TRIM5α by identifying HSV-1 as another large DNA virus that is recognized and restricted by this host factor. Previously, TRIM5α was shown to restrict retroviruses, flaviviruses and poxviruses [19,21,22]. The remarkable breadth of viral proteins that can be recognized by TRIM5α poses the questions of how is this possible and how is the antiviral activity mediated?

TRIM5α targets the HSV-1 capsid protein VP19C, which does not share recognizable primary amino acid sequence with other TRIM5α targets, such as the capsid proteins of HIV (CA), and VACV (L3). Furthermore, TRIM5α also co-precipitated strongly with VP19C orthologs in other human herpesviruses despite low amino acid identity, suggesting that TRIM5α recognition does not depend on sequence conservation. The ability to recognize VP19C requires the C-terminal PRY-SPRY domain of TRIM5α, however, this domain alone was not sufficient to co-precipitate with VP19C and co-precipitation required TRIM5α dimerization, mediated by the coiled-coil domain. Similarly, the recognition of the retroviral CA and poxvirus L3 capsid proteins also requires the TRIM5α coiled-coil and PRY-SPRY domain [22,44]. This sequence promiscuity supports a model in which TRIM5α recognizes repeated structures such as shared capsid geometries, or soluble capsid proteins rather than primary sequence. Most other pattern recognition receptors (PRRs) for viral pathogen-associated molecular patterns (PAMPs) recognize viral nucleic acids that differ in either structure or location from cellular nucleic acids. TRIM5α exemplifies a different type of PRR that recognizes viral proteins that can multimerize, others include MxA (bunyaviruses [45] and influenza viruses [46]), MxB (herpesviruses [47] and retroviruses [48–50]) and TRIM11 (retroviruses [51]).

In addition to the PRY-SPRY and coiled-coil domains, the anti-HSV-1 activity of TRIM5α also requires the E3 ubiquitin ligase activity, and more specifically, the ability to synthesize unanchored polyubiquitin chains. Whereas the catalytically dead L19R mutant was no longer antiviral, the partially defective mutant N70A, which retains its ability to generate unanchored polyubiquitin chains, restricted HSV-1 replication. This contrasts with TRIM5α-mediated restriction of poxviruses, where synthesis of anchored, but not unanchored, chains is essential.

The precise mechanism by which TRIM5α restricts HSV-1 through its interaction with VP19C, whether affecting VP19C intracellular trafficking or its function during capsid protein nuclear entry, assembly and egress, or activation of innate immunity, remains to be determined. However, in this first report of TRIM5α-mediated restriction of HSV-1, we have gone some distance to understanding it. Specifically, TRIM5α strengthened VP19C-VP23, but not VP19C-VP5, interaction in an E3 ubiquitin ligase activity-dependent manner, although VP19C ubiquitylation was not observed under the conditions tested. The accumulation of VP19C and VP23 in the nuclei of infected cells expressing wild-type TRIM5α, but not the catalytically inactive mutant, suggests that TRIM5α may interfere with capsid protein nuclear import, capsid assembly or nuclear egress. Moreover, the observation that TRIM5α itself accumulates in the perinuclear region at late stages of infection would be consistent with a role in affecting capsid protein transport or viral morphogenesis. Future detailed investigation by electron microscopy to visualize capsid assembly, maturation or nuclear export in the presence or absence of TRIM5α or mutants thereof might answer this question. Although TRIM5α enhances, rather than prevents, specific interactions of HSV-1 capsid proteins, this may well be antiviral. A parallel exists with orthopoxviruses, where the two anti-viral drugs tecovirimat and G243-1720, promote stabilization of protein F13 (orthopoxvirus gene OPG57) dimers to inhibit its function in enabling wrapping of intracellular virions and their egress from the cell [52,53]. A caveat to these observations is that while we recognize the importance of studying viral proteins expressed at endogenous levels, we were restricted by the lack of antibodies against the herpesvirus capsid proteins VP19C and VP23. Therefore, the influence of TRIM5α on VP19C and its binding partners was investigated by over-expressing epitope-tagged VPs during HSV-1 infection and in cells that do or do not express endogenous TRIM5, so that the conditions tested would be as close to natural infection as possible.

In addition to these modifications to HSV-1 capsid protein interactions and localization, and in line with prior reports linking TRIM5α to innate immune signaling [19], we observed that TRIM5α synergized with VP19C to enhance NF-κB activation. A response was also elicited, albeit less potently, by VP19C in the absence of TRIM5α, indicating that VP19C is sensed by at least one additional host factor that links to NF-κB activation, with TRIM5α either amplifying this signal or providing an independent signal.

### Viral evasion strategies

The persistent selective pressure of TRIM5α as a restriction factor for multiple types of virus is also supported by the observation that several viruses have evolved countermeasures to minimize the antiviral activity of TRIM5α. HSV-1 induces proteasomal degradation of TRIM5α [24,26] through a yet-to-be identified mechanism independent of TRIM5α autoubiquitylation. This degradation was induced by multiple HSV-1 strains and in different cell types, including neuronal cells. Poxviruses also induce proteasomal degradation of TRIM5α and the viral protein responsible was identified as OPG29 (C6), a small Bcl-2-like protein that is conserved in orthopoxviruses including variola virus and monkeypox virus [22].

The identification of the viral protein(s) responsible for TRIM5α degradation was attempted. ICP0 was the primary candidate because co-localization of ICP0 and TRIM5α in HSV-1 infected cells was noted [26] and ICP0 itself is an E3 ligase [54,55]. However, interaction between TRIM5α and ICP0 by affinity purification was not observed under the conditions tested. Furthermore, infection by a virus lacking ICP0 (v∆ICP0) in cell lines inducibly expressing ICP0 was carried out to compare TRIM5α abundance with or without ICP0 expression. The result was equivocal in that there was a slight increase in TRIM5α in the absence of ICP0, but still less compared with mock infection. So ICP0 might have a small role, but we could not conclude that ICP0 is solely responsible for TRIM5α degradation and further investigation is needed in the future.

As a second strategy, poxviruses, retroviruses and herpesviruses also recruit CypA into virus particles via interaction with the same capsid protein that TRIM5α binds to [22,23,40]. In each case, CypA is proviral, and this is lost in CypA mutants lacking prolyl isomerase activity. But the precise proviral mechanism differs for different viruses. Like TRIM5α, CypA also accumulated to the perinuclear region late during HSV-1 infection. Co-immunoprecipitation mapped its interaction with the capsid protein VP19C to the C terminus and CypA also interacted with VP19C orthologs in other human herpesviruses. Rather surprisingly, despite the similar localization and interaction with the virus capsid protein, CypA and TRIM5α act through distinct, non-overlapping mechanisms. CypA enhanced HSV-1 replication independent of TRIM5α, perhaps by promoting capsid maturation or nuclear egress, and did not affect VP19C–VP23 interaction or localization. This separation of activity reveals an additional layer of host–virus co-evolution centered on capsid-associated host factors. So for retroviruses and poxviruses, the pro-viral activity of CypA is dependent on the presence of TRIM5α [22,23,40], but for HSV-1, it is independent of TRIM5α, at least under the conditions tested. Similarly, in retroviruses and poxviruses, the antiviral activity of CsA and non-immunosuppressive derivatives alisporivir and NIM811, which target CypA, is TRIM5α-dependent. In contrast, for HSV-1, CsA is antiviral even in the absence of TRIM5α. However, it is worth noting that CypA was also thought to function independent of TRIM5α for HIV-1 until experiments were performed in primary human blood cells [56], therefore, further assessment of the interdependency between TRIM5α, CypA and CsA in primary cells is warranted.

The inhibitory effects of CsA, particularly its ability to suppress the replication of several HSV-1 strains in different cells, including neuronal cells, demonstrate the therapeutic potential of targeting CypA–capsid interactions. These findings raise the possibility that CypA-directed inhibitors could be repurposed or optimized as antiviral agents against HSV-1, providing a promising avenue for intervention in neuronal infections where treatment options remain limited. Moreover, given that CypA interacts with orthologs of VP19C in other human herpesviruses, CsA derivatives might have applications against herpesvirus infections more widely. Although CypA acts independently of TRIM5α under the conditions tested, the fact that both host factors engage with the same capsid protein emphasizes the significance of VP19C as a host–virus interaction interface and suggests that this region of the capsid represents an important target for drug development. Virus resistance to such drugs will be less likely to emerge, because the drug targets a host protein.

Taken together, these findings raise important questions from both evolutionary and structural perspectives. TRIM5α restricts viruses from four distinct families: retroviruses, flaviviruses, poxviruses, and herpesviruses. This suggests that TRIM5α may detect conserved higher-order architectural motifs, such as repeating lattice geometry or surface topology, rather than specific amino acid sequences. This broad specificity may have evolved under sustained selective pressure from diverse DNA and RNA viruses. Defining the consensus structural determinants that enable TRIM5α recognition presents an important direction for future work.

## Materials and Methods

### Cell lines and cell culture

Cell lines used in this study are: human fetal foreskin fibroblast cells immortalized with human telomerase (HFFF-TERTs; male), HeLa (American Type Culture Collection (ATCC) CCL-2), T-REx293 (Life Technologies), Vero (ATCC CRL-1586) and U-87 MG (a gift from Dr Rong Zhang, School of Basic Medical Sciences, Shanghai Medical College, Fudan University). All cell lines were cultured in DMEM (Gibco), supplemented with 10% FBS (PAN Biotech) and 50 μg/ml penicillin–streptomycin (Gibco). T-REx293-derived cells that were stably transfected with pcDNA4/TO plasmids were further supplemented with 10 μg/ml blasticidin (Thermo Fisher) and 100 μg/ml zeocin (Gibco). Knockout, complementation and over-expression cell lines used in this study (Table A in S1 Appendix) were generated and described in Zhao et al., 2023 [22].

### Virus stocks

The viruses used in this study are: HSV-1 s17, HFEM and sc16, which were gifts from Prof. Gillian Elliott, Department of Microbial Sciences, School of Biosciences, University of Surrey. Viruses were grown in Vero cells. The supernatant of infected cells was collected, and the cell debris was removed by centrifugation. HSV-1 s17 expressing the capsid protein VP26 as a GFP-fusion protein was introduced previously [57]. Virus titre was measured by plaque assay on Vero cells. HCMV was a gift from Prof. Michael Weekes, Cambridge Institute for Medical Research, University of Cambridge.

### Plasmids

The TRIM5α and CypA expression plasmids were generated and described in Zhao et al., 2023 [22]. Epitope-tagged HSV-1 VP19C, VP23 and VP5 were generated by PCR from extracted virus DNA and cloned into the pcDNA3 and pcDNA4/TO plasmids with DNA encoding HA and TAP tags at the 3′ or 5’ end. pcDNA3 plasmids expressing VP19C truncation mutants were generated by PCR from the pcDNA4/TO VP19C-TAP plasmid. pcDNA4/TO-based plasmids expressing the VACV-WR N1 and C1 have been described [22]. EBV BORF1 gene was amplified by PCR from DNA extracted from EBV transformed B cells (gifts from Prof. Tao Dong, MRC Human Immunology Unit, University of Oxford) [58], and inserted into the pcDNA4/TO vector. VZV ORF20 was subcloned into the pcDNA4/TO vector from pLenti6.3.ORF20-V5 (a kind gift from Prof. Jan Rehwinkel, Radcliffe Department of Medicine, University of Oxford). KSHV ORF62 was amplified by PCR and subcloned into the pcDNA4/TO vector from pFastBac.ORF62 (a gift from Prof. Prashant Desai, School of Medicine, Johns Hopkins University). HCMV UL46 was amplified from DNA extracted from HCMV-infected cells and subcloned into the pcDNA4/TO. The NF-κB firefly luciferase reporter and *TK-Renilla* luciferase plasmids were gifts from A. Bowie (Trinity College Dublin, Republic of Ireland). For in vitro transcription and translation assays, *VP19C (UL38)–HA, N1-HA, TAP–TRIM5α* and *TAP–CypA (PPIA)* were amplified from pcDNA4/TO plasmids either described previously [22] or from the above-described expression vectors and cloned into the pF3A WG (BYDV) plasmid (Promega). For the construction of vectors expressing a protein with different tags (HA or TAP), restriction enzymes were used to digest the open reading frame without tag from one vector and subclone it into the other vector that encodes the other tag. Oligonucleotides and primers used for cloning and sequencing are listed in Table B in S1 Appendix. A complete list of plasmids is described in Table C in S1 Appendix.

### Virus replication, spread and infection assays

Cells used for virus growth assays were infected at either 0.01 or 5 plaque-forming units (pfu)/cell for low and high multiplicity of infection (MOI). At 2 hours post-infection (hpi), the inoculum was removed, cells were washed once with DMEM and cultured in DMEM containing 2% FBS. Cells were harvested at 24 hpi for 0.01 MOI and 12 hpi for 5 MOI. For assessment of the pharmacological inhibition of NF-κB activation on TRIM5-mediated HSV-1 restriction, cells were pretreated with 3 µM Takinib or DMSO for 12 h before infection at 0.01 pfu/cell. Samples were freeze-thawed three times and infectious viral titres were determined by plaque assay on Vero cells. To assess viral spread, T-REx293 or HeLa cell monolayers were infected with GFP-HSV-1 at 500 pfu/well and were overlaid with MEM 2% FBS supplemented with 2 mM l-glutamine, and 2% carboxymethylcellulose. Plaques were photographed at 24 hpi and the plaque size was measured using ImageJ. For immunoblotting, co-precipitation and immunofluorescence assays, cells were infected with the appropriate virus in DMEM–2% FBS.

### Transfection

Transfection was carried out following the protocol described previously [22]. In brief, transfection was carried out using either TransIT-LT1 reagent (Mirus Bio) for T-REx293 (for reporter gene and immunofluorescence assays) or polyethylenimine (PEI, Polysciences) for other assays. The required amount of plasmid DNA and TransIT-LT1 (Mirus Bio) or PEI (2 μl per 1 μg DNA) were mixed into Opti-MEM (Gibco) (50 μl per 1 μg DNA) and incubated at room temperature for 20 min. Culture medium was replenished with DMEM 2% FBS before transfection mixtures were added dropwise to cells.

### Immunoblotting

Immunoblotting was carried out following the protocol described previously [22]. Cells were scraped, washed with PBS (Sigma-Aldrich) and lysed with lysis buffer (50 mM Tris-HCl (pH 8.0), 150 mM NaCl, 1 mM EDTA, 10% (v/v) glycerol, 1% (v/v) Triton X-100 and 0.05% (v/v) NP-40), supplemented with protease (cOmplete Mini, Roche) and phosphatase (PhosSTOP, Roche) inhibitors. Cell lysates were clarified by centrifugation at 13,000*g* for 10 min at 4 °C. Laemmli buffer (5×) was added to the samples and boiled at 100 °C for 10 min. Equal volumes of protein samples were loaded onto SDS–polyacrylamide gels or NuPAGE 4–12% Bis-Tris precast gels (Invitrogen), separated by electrophoresis, and transferred to a nitrocellulose membrane (GE Healthcare). Membranes were blocked at room temperature with 5% (v/v) skimmed milk in TBS containing 0.1% (v/v) Tween-20 (TBS/T) for 1 h before incubation with the appropriate primary antibodies at room temperature for 1 h or at 4 °C overnight. After three 5-min washes with TBS/T, membranes were incubated with fluorophore-conjugated secondary antibodies (LI-COR Biosciences) at room temperature for 1 h, washed three times with TBS/T and left to dry before imaging. Band intensities indicated in immunoblots and graphs were quantified using the Image Studio software (LI-COR Biosciences) and normalized to protein levels of loading control (β-tubulin or histone H3). Primary and secondary antibodies used are listed in Table D in S1 Appendix.

### Co-precipitation assays

Co-precipitation assays were carried out following protocol described previously [22]. In brief, T-REx293 cells were seeded in 10-cm dishes for transfection with indicated epitope-tagged plasmids, or for transfection followed by infection with HSV-1 s17. Cells were harvested 24 h after transfection or 12 hpi on ice and washed with ice-cold PBS. For co-precipitation with Strep-Tactin Superflow agarose resin (IBA), 0.5% NP-40 in PBS was used as lysis and wash buffers. For immunoprecipitation with anti-HA agarose (Sigma-Aldrich), HA lysis/wash buffer (50 mM Tris-HCl [pH 6.8], 150 mM NaCl and 1% NP-40) was used. Lysis buffers were supplemented with protease (cOmplete Mini, Roche) and phosphatase (PhosSTOP, Roche) inhibitors. Lysis was carried out at 4 °C for 2 h. The insoluble fraction was collected by centrifugation at 13,000*g* for 15 min at 4 °C. Ten percent of the soluble fraction was collected as input and the remaining volume was incubated with the indicated resin at 4 °C overnight. Protein-bound resins were washed three times with ice-cold wash buffer and proteins were eluted by boiling in 2 × Laemmli buffer before analysis by SDS–PAGE and immunoblotting.

### Cell fractionation assay

T-REx293 cells expressing proteins of interest were harvested 24 h after transfection. Cells were washed with cold PBS twice before lysis with fractionation buffer (50 mM Tris-HCl (pH 8), 150 mM NaCl and 1% NP-40) supplemented with protease (cOmplete Mini, Roche) and phosphatase (PhosSTOP, Roche) inhibitors for 20 min on ice. The lysate was clarified by centrifugation at 13,000*g* for 10 min at 4 °C. The supernatant was collected. The insoluble fraction was washed once with fractionation buffer followed by centrifugation at 13,000*g* for 10 min at 4 °C. Equal volumes of Laemmli buffer was added to both fractions and nuclease was added to the insoluble fraction before analysis by SDS-PAGE and immunoblotting.

### In vitro transcription and translation

Equal amounts of pF3A-derived plasmids expressing proteins of interest were added to the TnT Sp6 high yield wheat germ protein expression system (Promega) according to the manufacturer’s instructions.

### Immunofluorescence

Immunofluorescence was carried out following the protocol described previously [22]. T-REx293-derived cell lines were seeded on poly-d-lysine (Sigma-Aldrich)-coated sterile glass coverslips in six-well plates. Cells were either transfected or induced with 150 ng/ml doxycycline (Melford) to express the protein of interest at least 12 h before infection at 5 pfu/cell with the appropriate GFP-HSV-1 for 10 h. To harvest, cells were washed twice with warm PBS and fixed with 4% (v/v) paraformaldehyde for 15 min. Samples were quenched with 150 mM ammonium chloride for 5 min, washed twice with PBS and permeabilized with 0.1% Triton X-100 in PBS for 5 min. Cells were blocked with 10% (v/v) FBS in PBS for 30 min followed by staining with primary antibodies in 10% (v/v) FBS in PBS for 1 h at room temperature. Coverslips were washed three times with 10% (v/v) FBS in PBS for 5 min each and incubated with the appropriate AlexaFluor fluorophore-conjugated secondary antibodies (Molecular Probes) diluted in 10% (v/v) FBS in PBS supplemented with 2.5% of the corresponding normal serum (donkey or goat; Sigma-Aldrich) for 30 min in the dark at room temperature. Coverslips were washed twice with 10% (v/v) FBS in PBS and once with PBS before mounting onto glass slides with Mowiol 4–88 (Calbiochem) containing 0.5 μg/ml 4′,6-diamidino-2-phenylindole (DAPI; Biotium). Images were acquired on Olympus FV3000 Confocal Microscope using FLUOVIEW software. The antibodies used in immunofluorescence are listed in Table D in S1 Appendix.

### Reporter gene assay

T-REx293 *TRIM5*^−/−^ cells were seeded in 96-well plates. Plasmids expressing tagged proteins were co-transfected with 100 ng NF-κB–luciferase and 10 ng *Renilla* luciferase reporter plasmids using TransIT-LT1 (Mirus Bio). Doxycycline (150 ng/mL) was added at the time of transfection for 24 h to induce protein expression. Cells were then harvested in passive lysis buffer (Promega). Firefly luciferase activity was measured and normalized to *Renilla* luciferase control. Fold induction was calculated relative to empty vector. Protein expression levels were determined by immunoblotting.

### Statistical analysis

Data are presented as means ± s.e.m., and statistical significance was analyzed in Prism (GraphPad) using Welch’s ANOVA test and followed by post-hoc Dunnett’s T3 multiple comparisons test where indicated or two-tailed unpaired *t*-test with Welch’s correction. The exact *P* values are shown in each figure, and the horizontal bars indicate the samples being compared. The number of repeats and the values of *n* in each experiment are indicated in the respective figure legends; *n* represents the number of biological replicates.

## Supporting information

S1 Appendix Table A. Cell lines used in this study. Table B. Oligonucleotide primers used in this study. Table C. Plasmids used in this study. Table D. Antibodies used in this study.
(DOCX)

S1 File S1 raw images. (PDF)
