# Supplementary material for: Independent proviral and antiviral host factors recognize the same capsid protein in divergent human herpesviruses
Source: PLoS Pathog. 2026 Jul 8;22(7):e1014376. doi: 10.1371/journal.ppat.1014376 (PMC13345423; doi:10.1371/journal.ppat.1014376)
Supplement: S1 Appendix — Cell lines used in this study. Table B. Oligonucleotide primers used in this study. Table C. Plasmids used in this study. Table D. Antibodies used in this study. (DOCX) [file ppat.1014376.s001.docx]

Table A. Cell lines used in this study.

| **Cell lines** | **Source** | **Identifier** |
| --- | --- | --- |
| Human fetal foreskin fibroblasts (HFFFs) immortalized with human telomerase (HFFF-TERTs) | Stanton et al., 2007 | N/A |
| HeLa (human cervical adenocarcinoma epithelial cell line) | ATCC | ATCC: CCL-2 |
| T-REx-293 | Life technologies | R71007 |
| VERO E6 | ATCC | ATCC: CRL-1586 |
| CRISPR Ctrl HeLa (for TRIM5-/-) | Zhao et al., 2023 | N/A |
| TRIM5-/- clone 1 HeLa | Zhao et al., 2023 | N/A |
| TRIM5-/- clone 2 HeLa | Zhao et al., 2023 | N/A |
| CRISPR RICE T-REx-293 (for TRIM5-/-) | Zhao et al., 2023 | N/A |
| TRIM5-/- clone 1 T-REx-293 | Zhao et al., 2023 | N/A |
| TRIM5-/- clone 2 T-REx-293 | Zhao et al., 2023 | N/A |
| EV T-REx-293 in T5-/- (complementation) | Zhao et al., 2023 | N/A |
| TAP-T5α WT T-REx-293 (complementation) | Zhao et al., 2023 | N/A |
| TAP-T5γ WT T-REx-293 (complementation) | Zhao et al., 2023 | N/A |
| TAP-T5δ WT T-REx-293 (complementation) | Zhao et al., 2023 | N/A |
| EV T-REx-293 (over-expression) | Zhao et al., 2023 | N/A |
| FLAG-T5α WT T-REx-293 (over-expression) | Zhao et al., 2023 | N/A |
| FLAG-T5γ WT T-REx-293 (over-expression) | Zhao et al., 2023 | N/A |
| FLAG-T5δ WT T-REx-293 (over-expression) | Zhao et al., 2023 | N/A |
| FLAG-T5α L19R T-REx-293 (complementation) | Zhao et al., 2023 | N/A |
| FLAG-T5α N70A T-REx-293 (complementation) | Zhao et al., 2023 | N/A |
| FLAG-T5α R119E T-REx-293 (complementation) | Zhao et al., 2023 | N/A |
| FLAG-T5α ΔSPRY T-REx-293 (complementation) | Zhao et al., 2023 | N/A |
| CRISPR RICE T-REx-293 (for CypA-/-) | Zhao et al., 2023 | N/A |
| CypA-/- clone 1 T-REx-293 | Zhao et al., 2023 | N/A |
| CypA-/- clone 2 T-REx-293 | Zhao et al., 2023 | N/A |
| EV T-REx-293 in CypA-/- (complementation) | Zhao et al., 2023 | N/A |
| TAP-CypA WT T-REx-293 (complementation) | Zhao et al., 2023 | N/A |
| TAP-CypA R55A T-REx-293 (complementation) | Zhao et al., 2023 | N/A |
| TAP-CypA F113A T-REx-293 (complementation) | Zhao et al., 2023 | N/A |
| CRISPR RICE T-REx-293 (for TRIM5-/- CypA-/-) | Zhao et al., 2023 | N/A |
| T5-/- CypA-/- clone 1 T-REx-293 | Zhao et al., 2023 | N/A |
| T5-/- CypA-/- clone 2 T-REx-293 | Zhao et al., 2023 | N/A |

Table B. Oligonucleotide primers used in this study.

| **Oligo names** | **Sequence 5' - 3'** |  |  |  |
| --- | --- | --- | --- | --- |
| pcDNA4-VP19CWT-TAP fw | AAAAAGCTTGCCACCATGAAGACCAATCCGCTACCCG |  |  |  |
| pcDNA4-VP19CWT-TAP rev | AAAGCGGCCGCCGCGCATGCCC |  |  |  |
| pcDNA4-BORF1-HA fw | AAAAAGCTTATGTACCCATACGATGTTCCAGATTACGCTGC  GGCCGCGAAGGTCCAGGGGTCC |  |  |  |
| pcDNA4-BORF1-HA rev | AAATCTAGACTAGAGAATCACCTCCCAGTCAGAGACGTTGG  AATAAAAGCTGTCAATCTCCACCACG |  |  |  |
| pcDNA4-UL46-HA fw | TAAGCTTGCCACCATGGACGCGCGCGCGGTGGCCAAAC |  |  |  |
| pcDNA4-UL46-HA rev | ACGCGGCCGCGACGAATTCTCGAAAG |  |  |  |
| pcDNA4-ORF20-HA fw | GCAAGCTTGCCGCCATGGGGAGTCAAC |  |  |  |
| pcDNA4-ORF20-HA rev | ATGCGGCCGCATAATAACATTCGTTCC |  |  |  |
| pcDNA4-ORF62-HA fw | GCAAGCTTGCCACCATGAAGGTGCAG |  |  |  |
| pcDNA4-ORF62-HA rev | ATGCGGCCGCCAGAAACACAGTC |  |  |  |
| pcDNA3-VP19CC1-HA fw | AAAGGTACCGCCACCATGCATCTAACCCGCCAAGTGATCCTG |  |  |  |
| pcDNA3-VP19CC1-HA rev | TTTCGCGGCCGCCGCGCATGCCC |  |  |  |
| pcDNA3-HA-VP19CC2 fw | AAAGCGGCCGCGAAGACCAATCCGCTACCCG |  |  |  |
| pcDNA3-HA-VP19CC2 rev | AAATCTAGATTACCCGGCGCACACGGCGGT |  |  |  |
| pcDNA3-VP23-HA fw | AAAGGTACCGCCACCATGCTGGCGGACG |  |  |  |
| pcDNA3-VP23-HA rev | TTTCGCGGCCGCGGGATAGCGTATAAC |  |  |  |
| pcDNA3-VP5-HA fw | AAAGGTACCGCCACCATGGCCGCTCCCAA |  |  |  |
| pcDNA3-VP5-HA rev | TTTCGCGGCCGCCAGAGCCAGTCCCTT |  |  |  |
| pF3A-UL38-HA fw | TTTCGATCGGCCACCATGAAGACCAAT |  |  |  |
| pF3A-UL38-HA rev | TTTGTTTAAACTCAAGCGTAATCTGGAACATCG |  |  |  |

Table C. Plasmids used in this study.

| **Plasmid** | **Source** |
| --- | --- |
| pcDNA4/TO EV | Invitrogen |
| pcDNA4-TAP-T5α | Zhao et al., 2023 |
| pcDNA4-TAP-T5α ΔSPRY | Zhao et al., 2023 |
| pcDNA4-TAP-T5α RING | Zhao et al., 2023 |
| pcDNA4-TAP-T5α RB | Zhao et al., 2023 |
| pcDNA4-TAP-T5α BCS | Zhao et al., 2023 |
| pcDNA4-TAP-T5α CS | Zhao et al., 2023 |
| pcDNA4-TAP-CypA WT | Zhao et al., 2023 |
| pcDNA4-TAP-CypA R55A | Zhao et al., 2023 |
| pcDNA4-TAP-CypA F113A | Zhao et al., 2023 |
| pcDNA4-TAP-coN1 | Maluquer de Motes et al., 2014 |
| pcDNA4-VP19CWT-TAP | this paper |
| pcDNA4-HA-BORF1 | this paper |
| pcDNA4-HA-ORF20 | this paper |
| pcDNA4-HA-UL46 | this paper |
| pcDNA4-HA-ORF62 | this paper |
| pcDNA3 EV | Invitrogen |
| pcDNA3-HA-coL3 | Zhao et al., 2023 |
| pcDNA3-HA-C1 | Zhao et al., 2023 |
| pcDNA3-HA-N1 | Zhao et al., 2023 |
| pcDNA3-VP19CWT-HA | this paper |
| pcDNA3-VP19CC1-HA | this paper |
| pcDNA3-HA-VP19CC2 | this paper |
| pcDNA3-VP5-HA | this paper |
| pcDNA3-VP23-HA | this paper |
| pF3A WG (BYDV) | Promega |
| pF3A-TAP-T5α | Zhao et al., 2023 |
| pF3A-TAP-CypA | Zhao et al., 2023 |
| pF3A-HA-VACVN1 | Zhao et al., 2023 |
| pF3A-VP19CWT-HA | this paper |

| **Antibody** | **Source** | **Identifier** |
| --- | --- | --- |
| Mouse anti-FLAG | Sigma-Aldrich | Cat# F3165; RRID:AB_259529 |
| Rabbit anti-FLAG | Sigma-Aldrich | Cat# F7425; RRID:AB_439687 |
| Mouse anti-HA | BioLegend | Cat# 901513; RRID:AB_2565335 |
| Rabbit anti-HA | Sigma-Aldrich | Cat# H6908; RRID:AB_260070 |
| Mouse anti-GAPDH | Sigma-Aldrich | Cat# G8795; RRID:AB_1078991 |
| Rabbit anti-α-actin | Sigma-Aldrich | [Cat# A2066; RRID:AB_476693](http://antibodyregistry.org/AB_476693) |
| Mouse anti-α-tubulin | Cell Signaling Technology | Cat# 3873; RRID:AB_1904178 |
| Rabbit anti-histone H3 | Cell Signaling Technology | Cat# 4499; RRID:AB_10544537 |
| Mouse anti-TRIM5 | Santa Cruz Biotechnology | [Cat# sc-373864; RRID:AB_10918111](http://antibodyregistry.org/AB_10918111) |
| Mouse anti-ICP0 | Santa Cruz Biotechnology | [Cat# sc-53070; RRID:AB_673704](http://antibodyregistry.org/AB_2116791) |
| Mouse anti-UL42 | Santa Cruz Biotechnology | Cat# sc-53331; RRID:AB_675635 |
| Rabbit anti-CypA | Invitrogen | Cat# PA1-025; RRID:AB_2169124 |
| IRDye 680RD-conjugated  goat anti-rabbit IgG | LI-COR | Cat# 926-68071; RRID:AB_10956166 |
| IRDye 800CW-conjugated  goat anti-mouse IgG | LI-COR | Cat# 926-32210; RRID:AB_621842 |
| Goat anti-mouse IgG (H+L)  secondary antibody, Alexa Fluor 488 | Invitrogen | Cat# A11001; RRID:AB_2534069 |
| Donkey anti-rabbit IgG (H+L)  secondary antibody, Alexa Fluor 546 | Invitrogen | Cat# A11010; RRID:AB_2534077 |

Table D. Antibodies used in this study.

**Reference**

1. Stanton, R. J. et al. Cytomegalovirus destruction of focal adhesions revealed in a high-throughput Western blot analysis of cellular protein expression. J Virol 81, 7860-7872, doi:10.1128/JVI.02247-06
2. Maluquer de Motes, C., Schiffner, T., Sumner, R. P. & Smith, G. L. Vaccinia virus virulence factor N1 can be ubiquitylated on multiple lysine residues. J Gen Virol 95, 2038-2049, doi:10.1099/vir.0.065664-0 (2014).
