# Supplementary material for: Independent proviral and antiviral host factors recognize the same capsid protein in divergent human herpesviruses
Source: PLoS Pathog. 2026 Jul 8;22(7):e1014376. doi: 10.1371/journal.ppat.1014376 (PMC13345423; doi:10.1371/journal.ppat.1014376)
Supplement: S1 File — (PDF) [file ppat.1014376.s002.pdf]

Fig1b

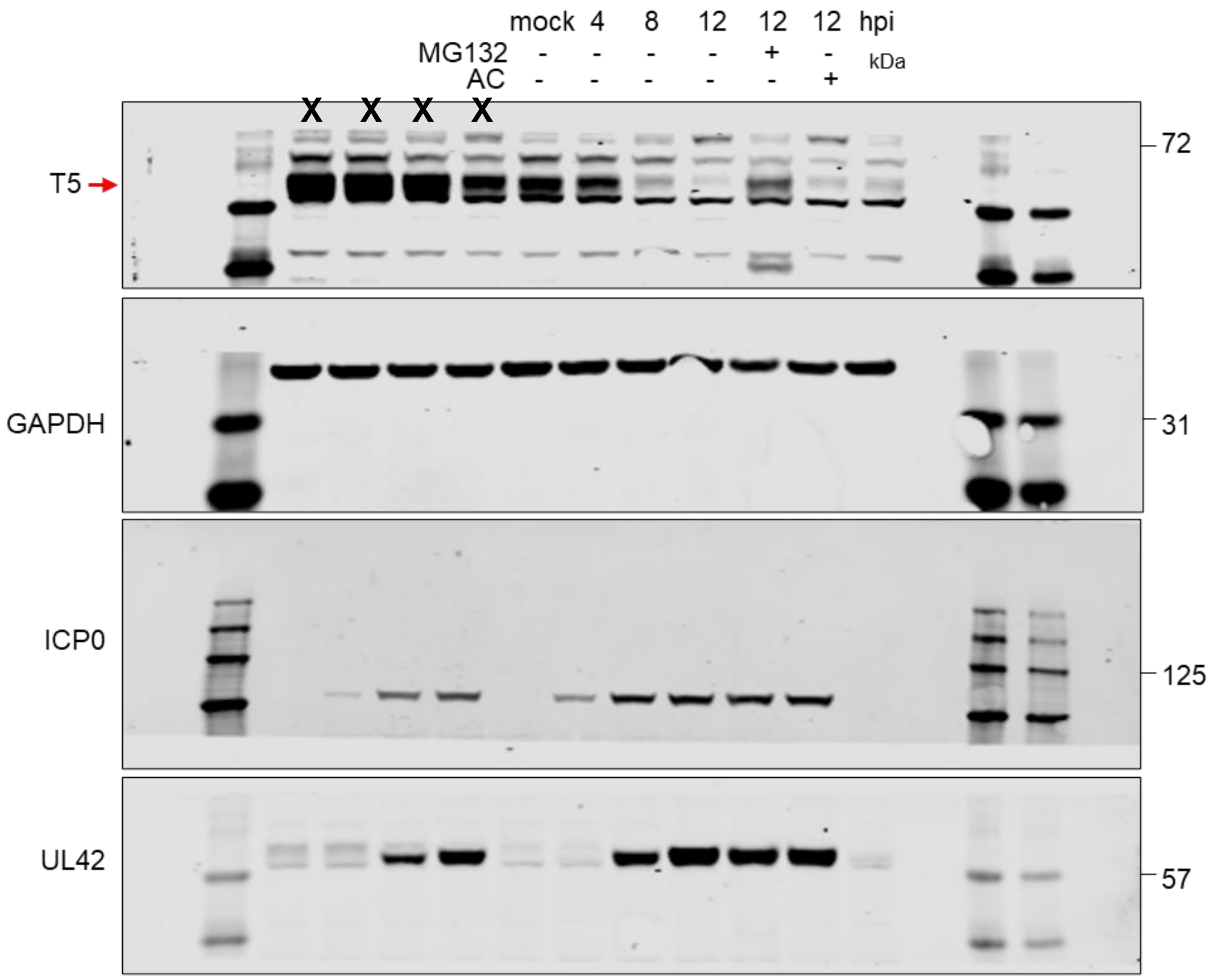

mock 4 8 12 12 12 hpi
MG132 - - - - + -
AC - - - - - +
kDa
X X X X
T5
GAPDH
ICP0
UL42
72
31
125
57

Fig1c

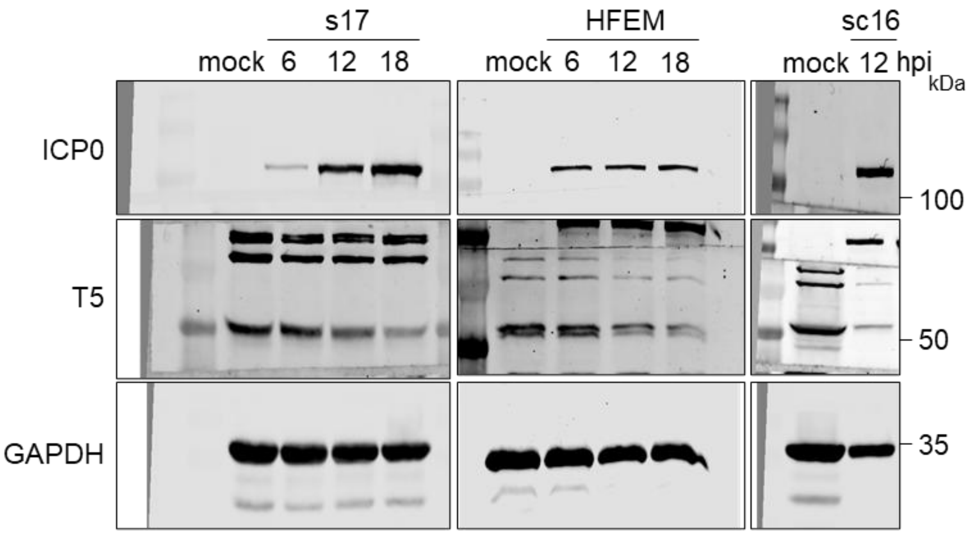

s17
HFEM
sc16
mock
6
12
18
mock
6
12
18
mock
12
hpi
kDa
ICP0
T5
GAPDH
100
50
35

Fig1d

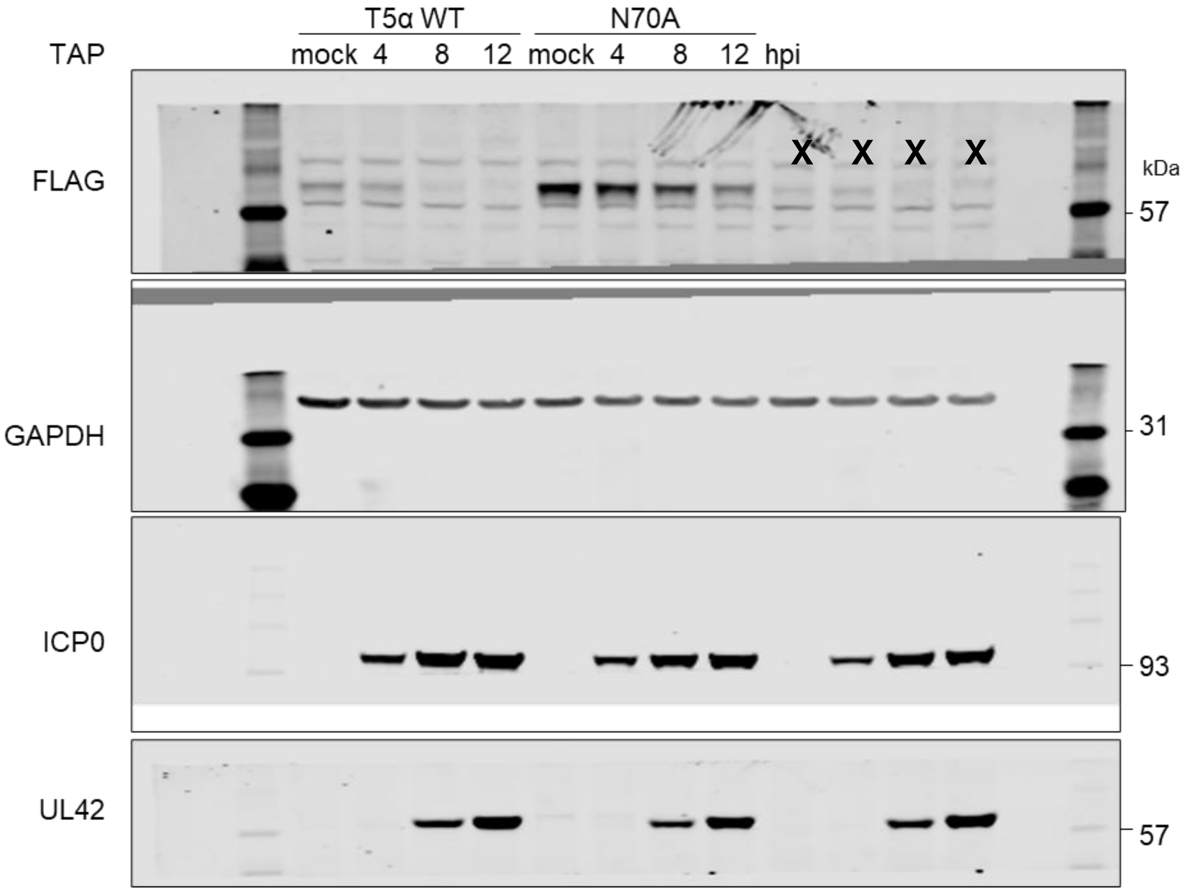
T5α WT
N70A
TAP
mock 4 8 12 mock 4 8 12 hpi
FLAG
X X X X
kDa
57
GAPDH
31
ICP0
93
UL42
57

Fig3a

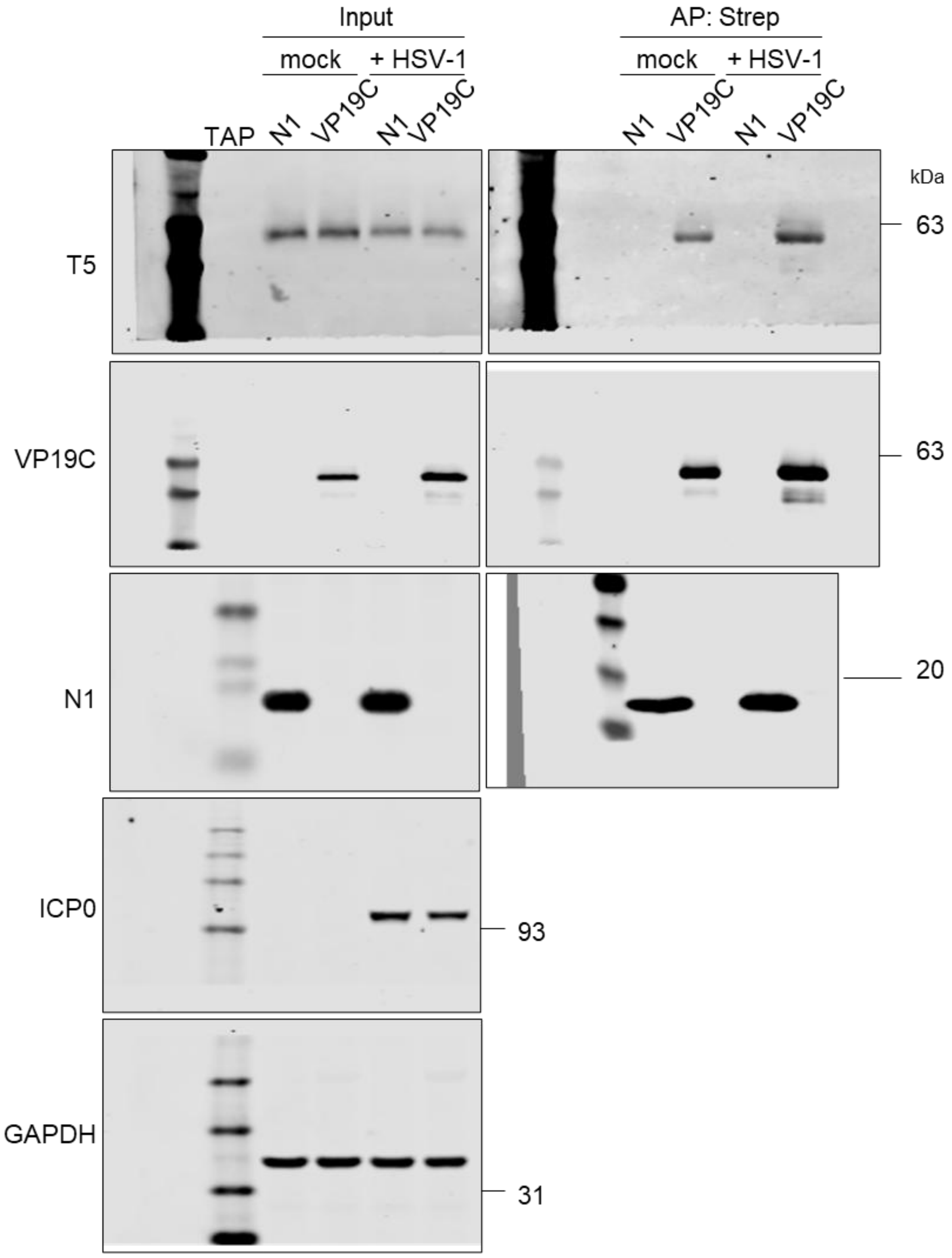

Input
mock
+ HSV-1
AP: Strep
mock
+ HSV-1
TAP
N1
VP19C
N1
VP19C
N1
VP19C
N1
VP19C
kDa
T5
63
VP19C
63
N1
20
ICP0
93
GAPDH
31

Fig3b

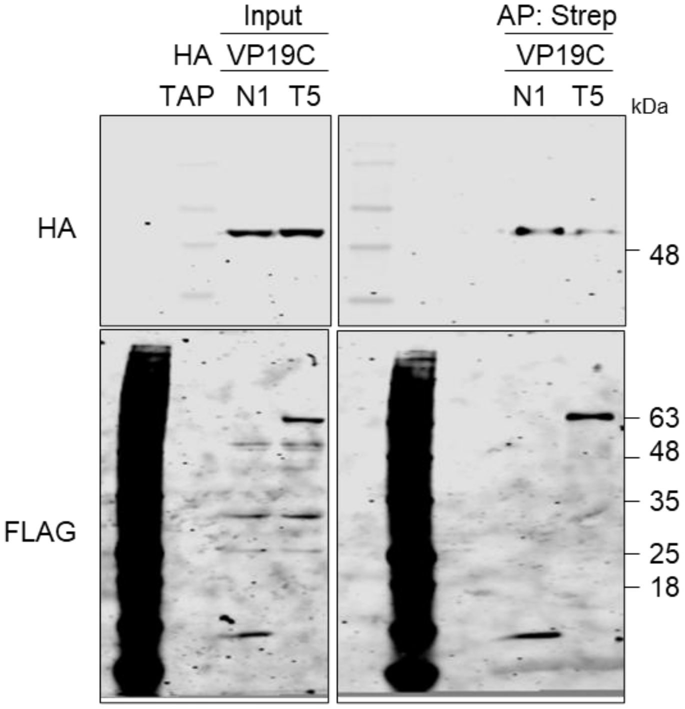
Input
AP: Strep
HA
VP19C
VP19C
TAP
N1
T5
N1
T5
kDa
HA
48
FLAG
63
48
35
25
18

Fig3c

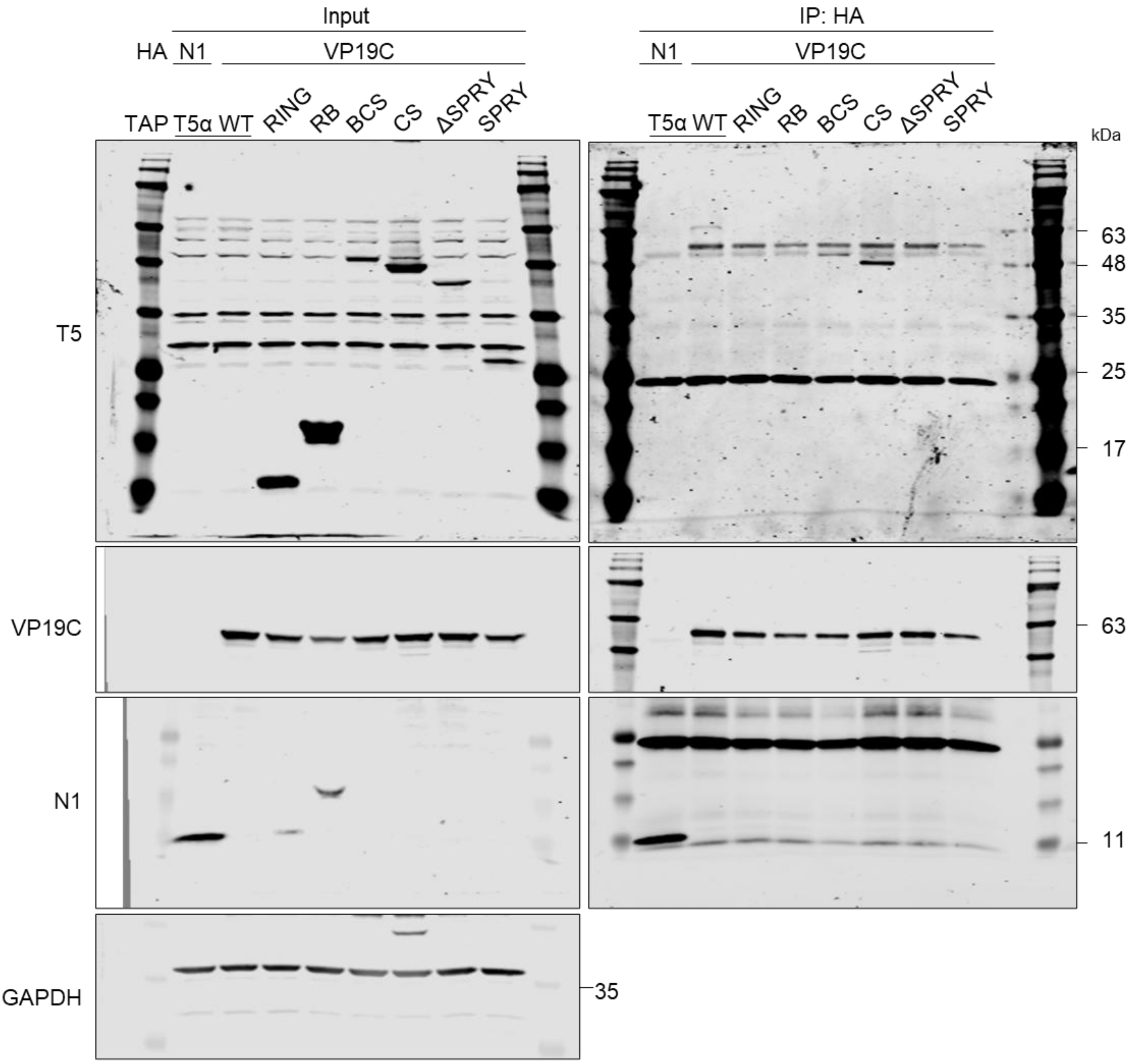
Input
IP: HA
HA
N1
VP19C
TAP
T5α WT
RING
RB
BCS
CS
ΔSPRY
SPRY
kDa
T5
VP19C
N1
GAPDH
63
48
35
25
17
63
11
35

Figd

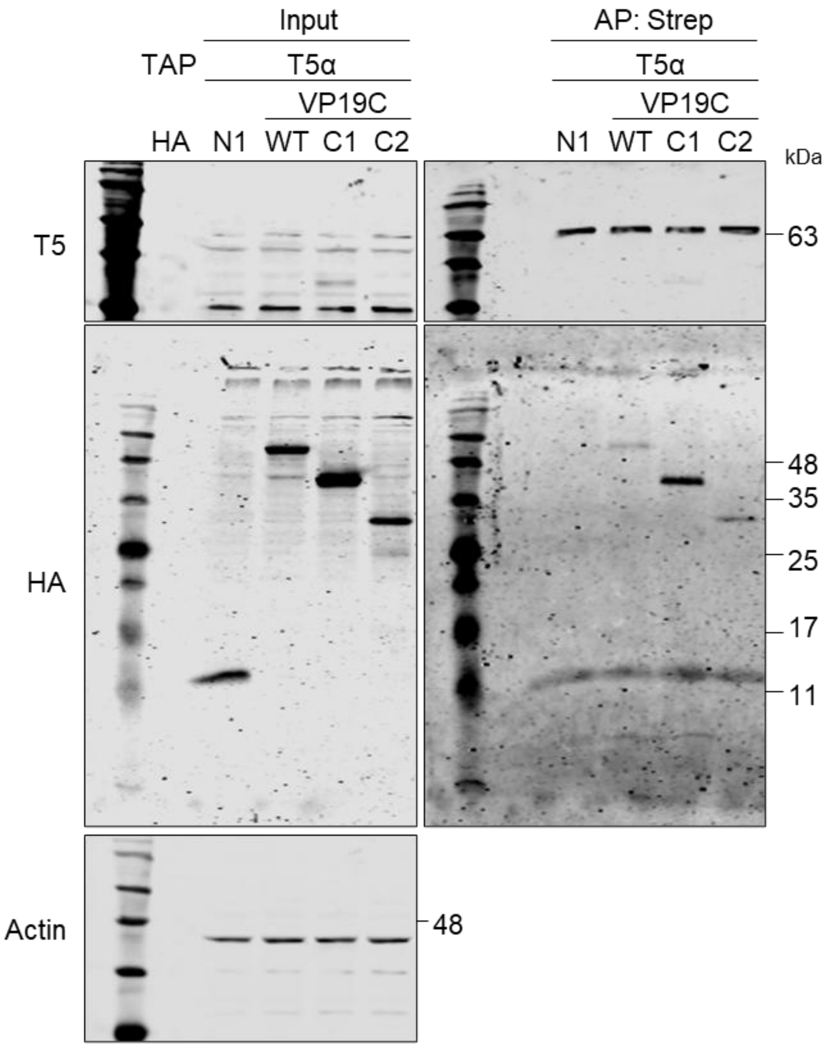

Input
AP: Strep
TAP
T5α
T5α
VP19C
VP19C
HA
N1
WT
C1
C2
N1
WT
C1
C2
kDa
T5
63
HA
48
35
25
17
11
Actin
48

Fig3e

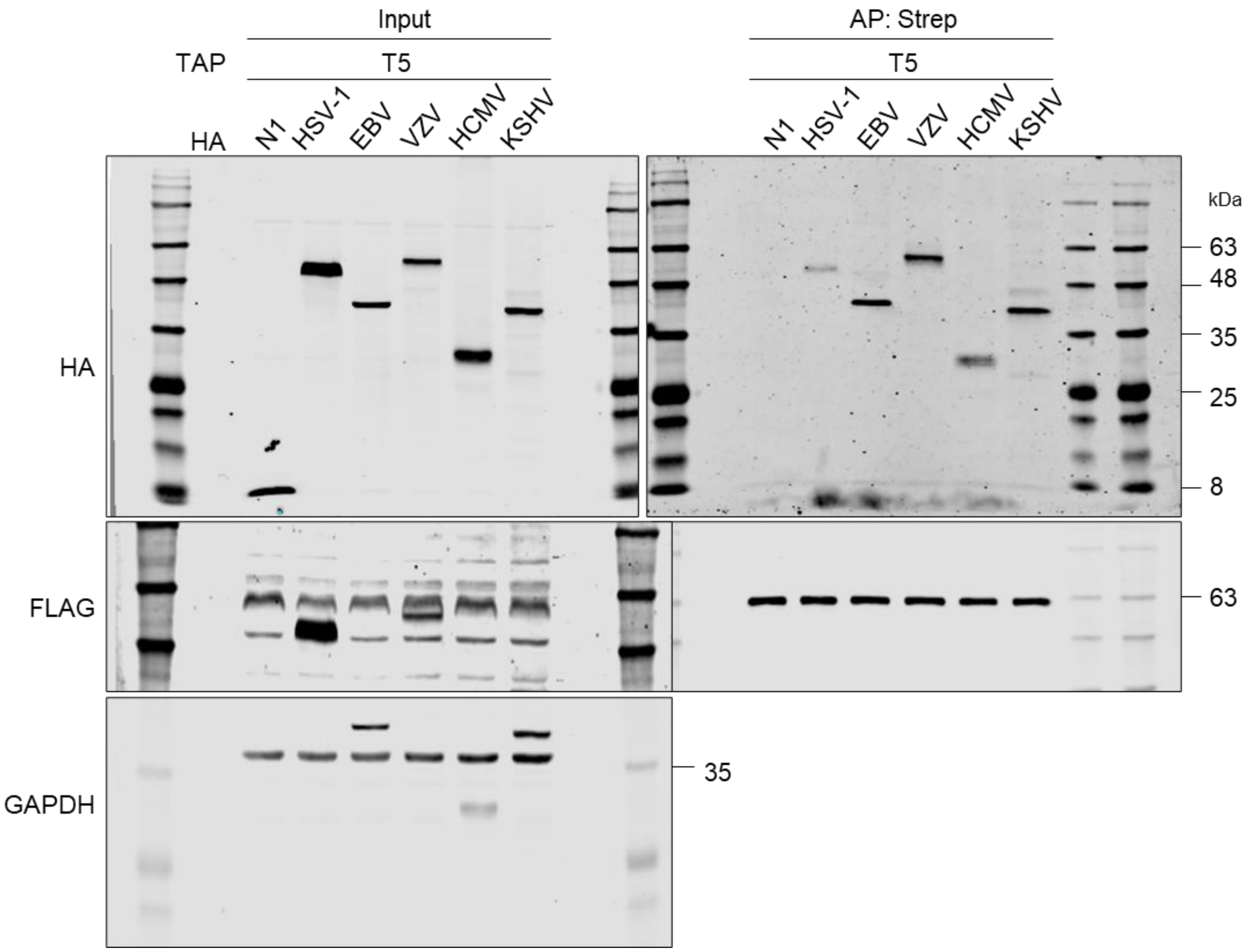

Input
AP: Strep
TAP
T5
T5
HA
N1
HSV-1
EBV
VZV
HCMV
KSHV
N1
HSV-1
EBV
VZV
HCMV
KSHV
kDa
63
48
35
25
8
HA
FLAG
63
GAPDH
35

Fig5a

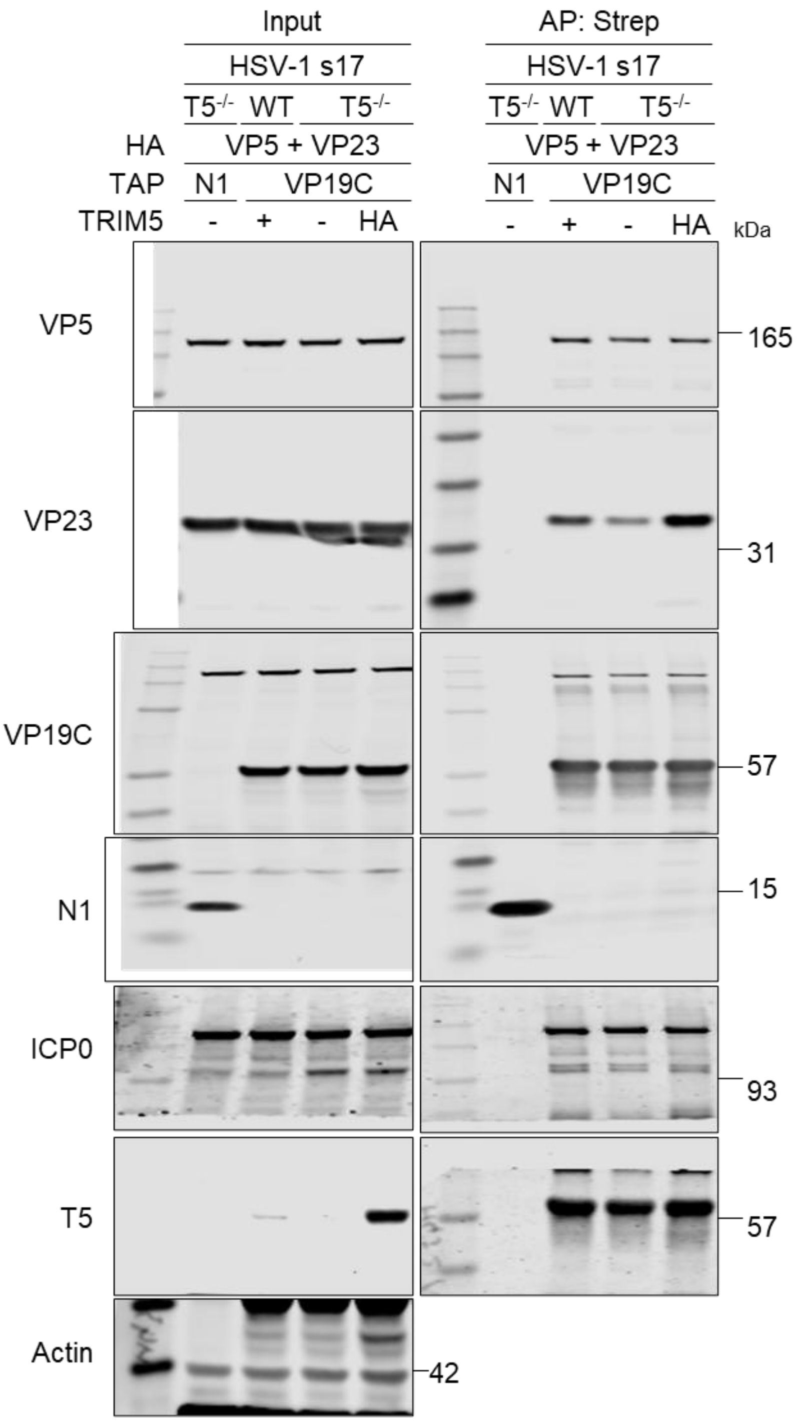
Input
AP: Strep
HSV-1 s17
HSV-1 s17
T5-/- WT T5-/-
T5-/- WT T5-/-
HA VP5 + VP23
VP5 + VP23
TAP N1 VP19C
N1 VP19C
TRIM5 - + - HA
- + - HA
kDa
VP5
165
VP23
31
VP19C
57
N1
15
ICP0
93
T5
57
Actin
42

Fig5b

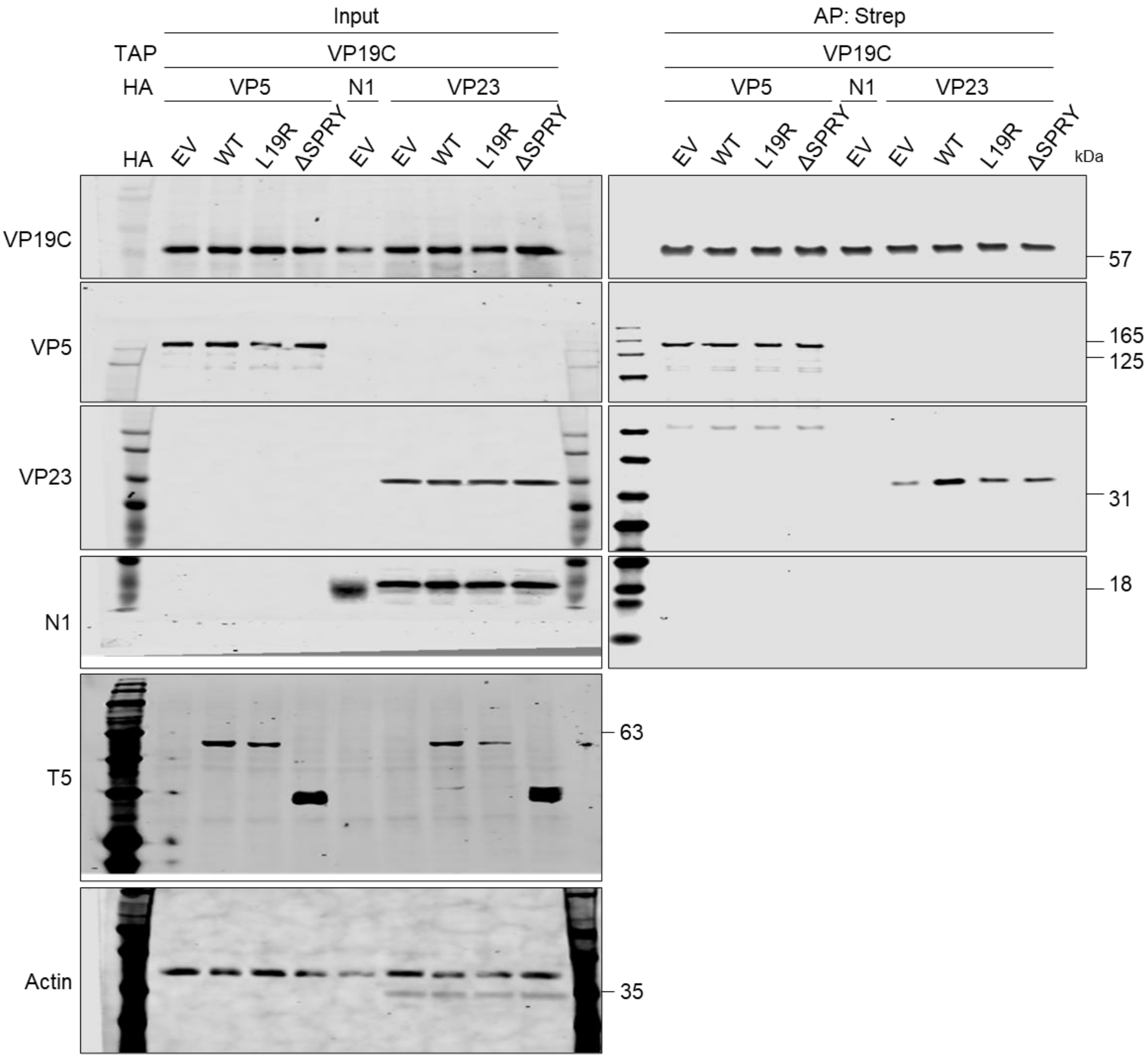
Input
AP: Strep
TAP
VP19C
HA
VP5
N1
VP23
HA
EV
WT
L19R
ΔSPRY
kDa
VP19C
VP5
VP23
N1
T5
Actin
57
165
125
31
18
63
35

Fig5c

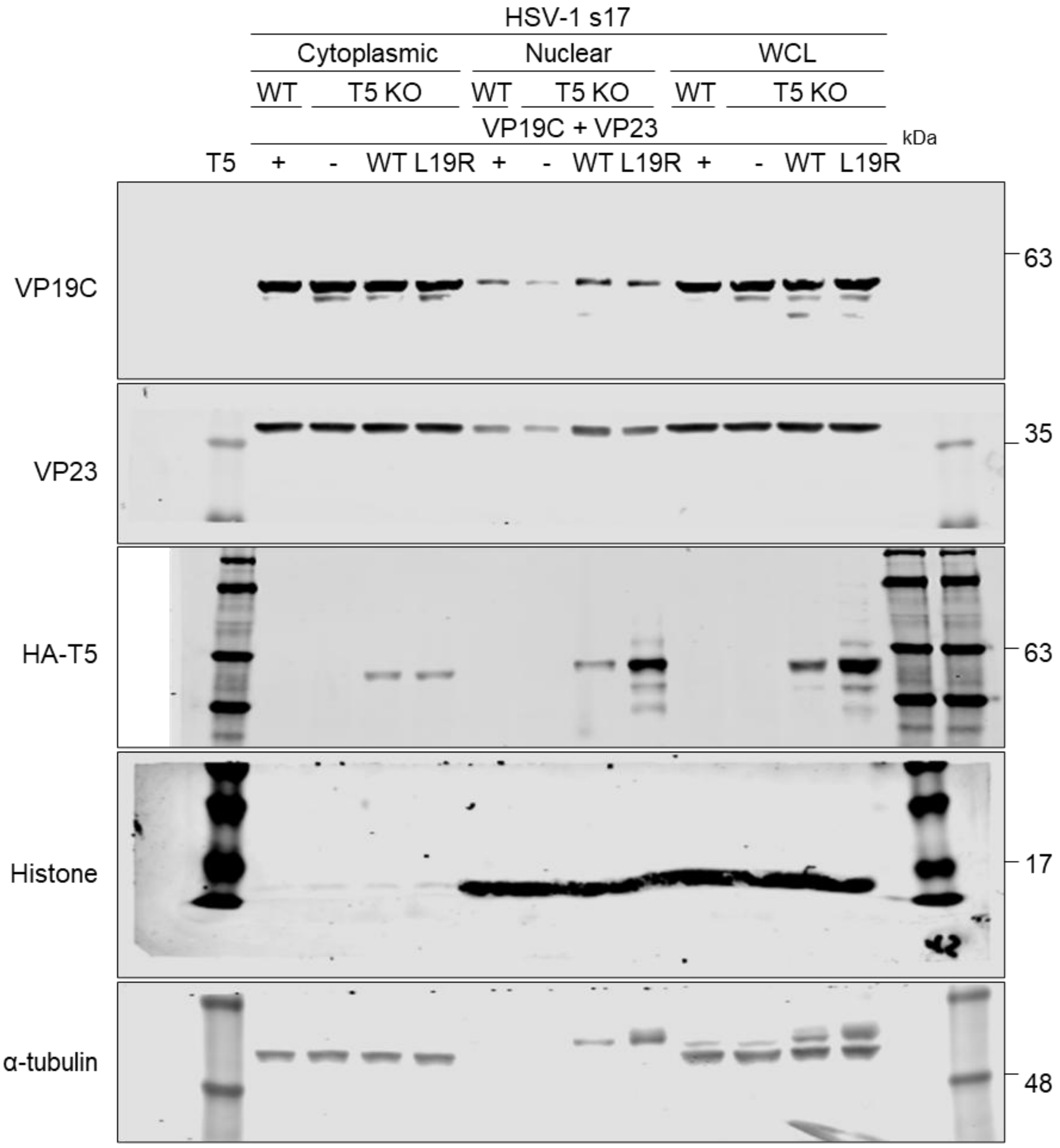
HSV-1 s17
Cytoplasmic
Nuclear
WCL
WT
T5 KO
WT
T5 KO
WT
T5 KO
VP19C + VP23
kDa
T5
+
-
WT
L19R
+
-
WT
L19R
+
-
WT
L19R
VP19C
63
VP23
35
HA-T5
63
Histone
17
α-tubulin
48

Fig5d

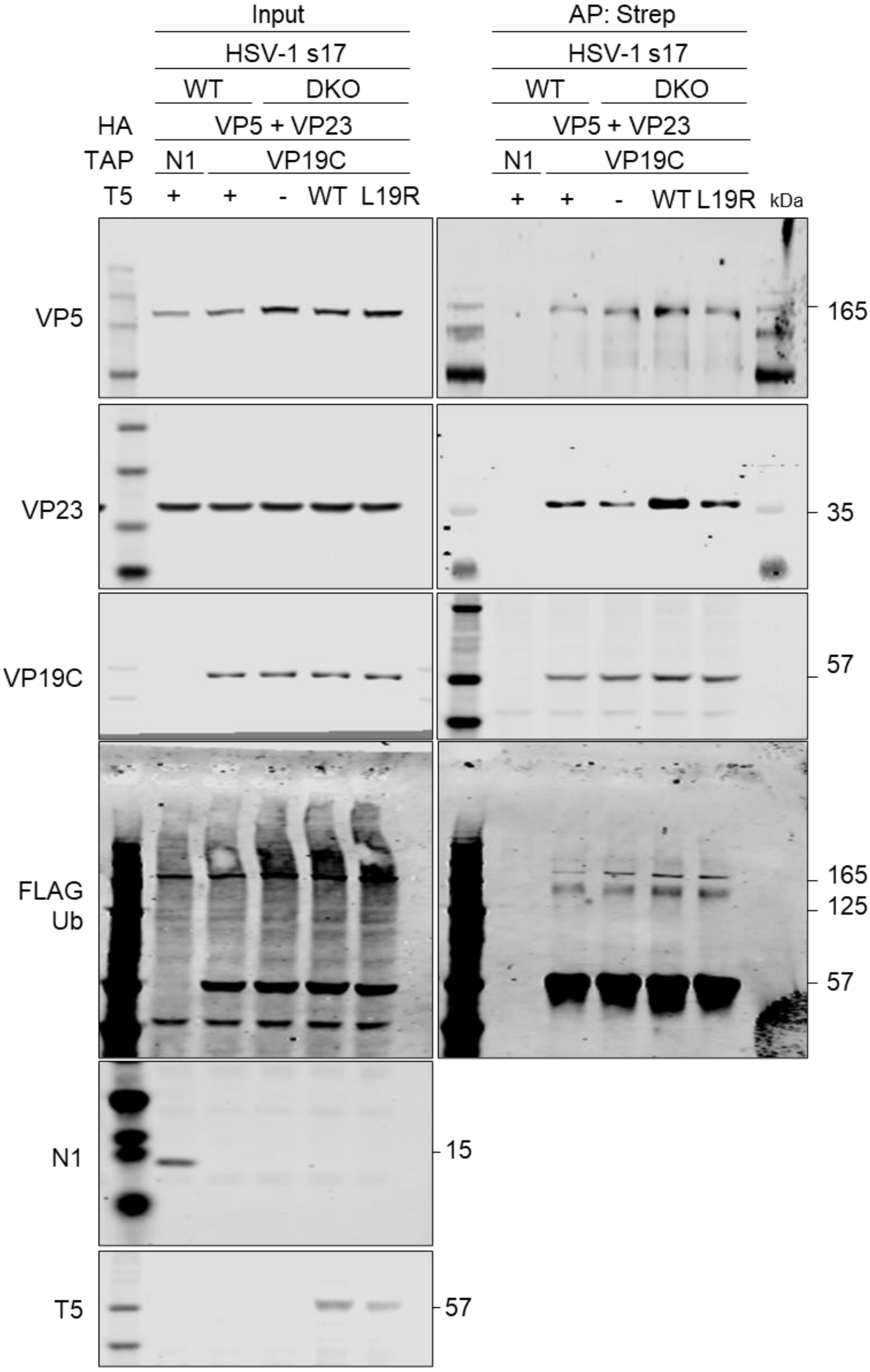

Input
AP: Strep
HSV-1 s17
HSV-1 s17
WT
DKO
WT
DKO
HA
VP5 + VP23
VP5 + VP23
TAP
N1
VP19C
N1
VP19C
T5
+
+
-
WT
L19R
+
+
-
WT
L19R
kDa
VP5
165
VP23
35
VP19C
57
FLAG
Ub
165
125
57
N1
15
T5
57

Fig5e

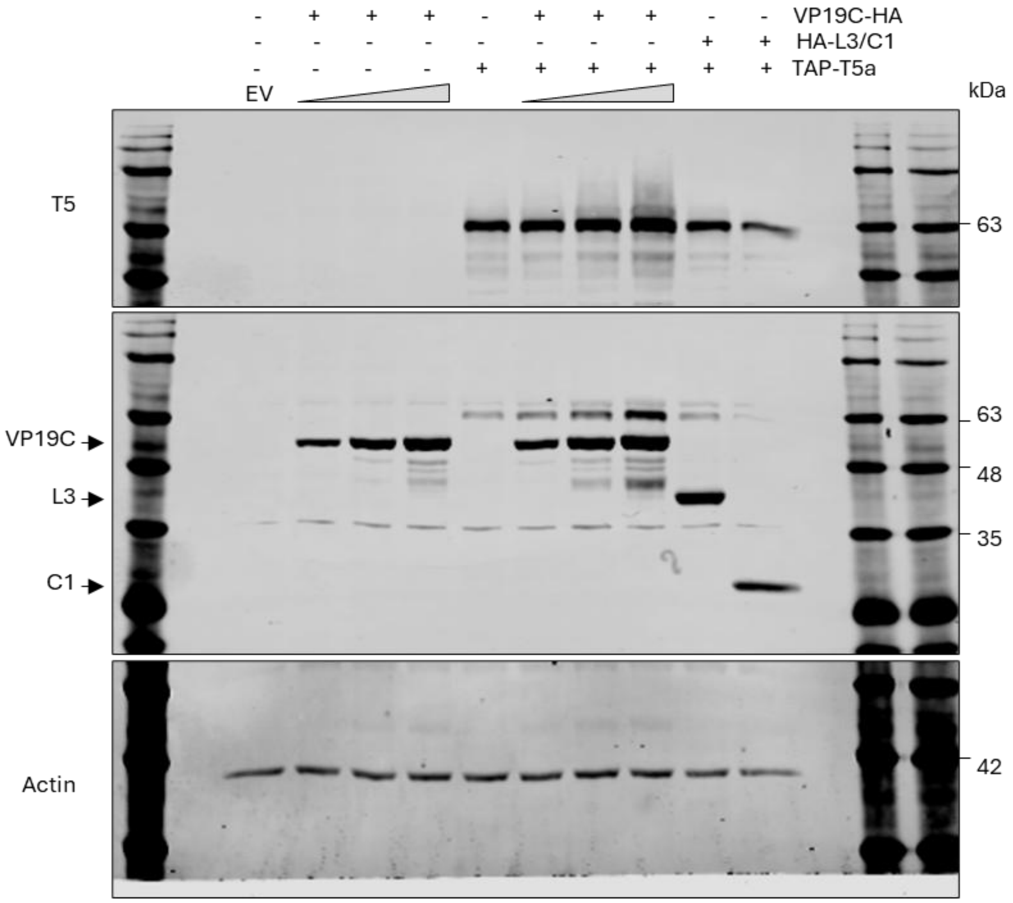
- + + + - + + + - - VP19C-HA
- - - - - - - - + + HA-L3/C1
- - - - + + + + + + TAP-T5a
EV
kDa
T5
63
VP19C
L3
C1
63
48
35
Actin
42

Fig8a

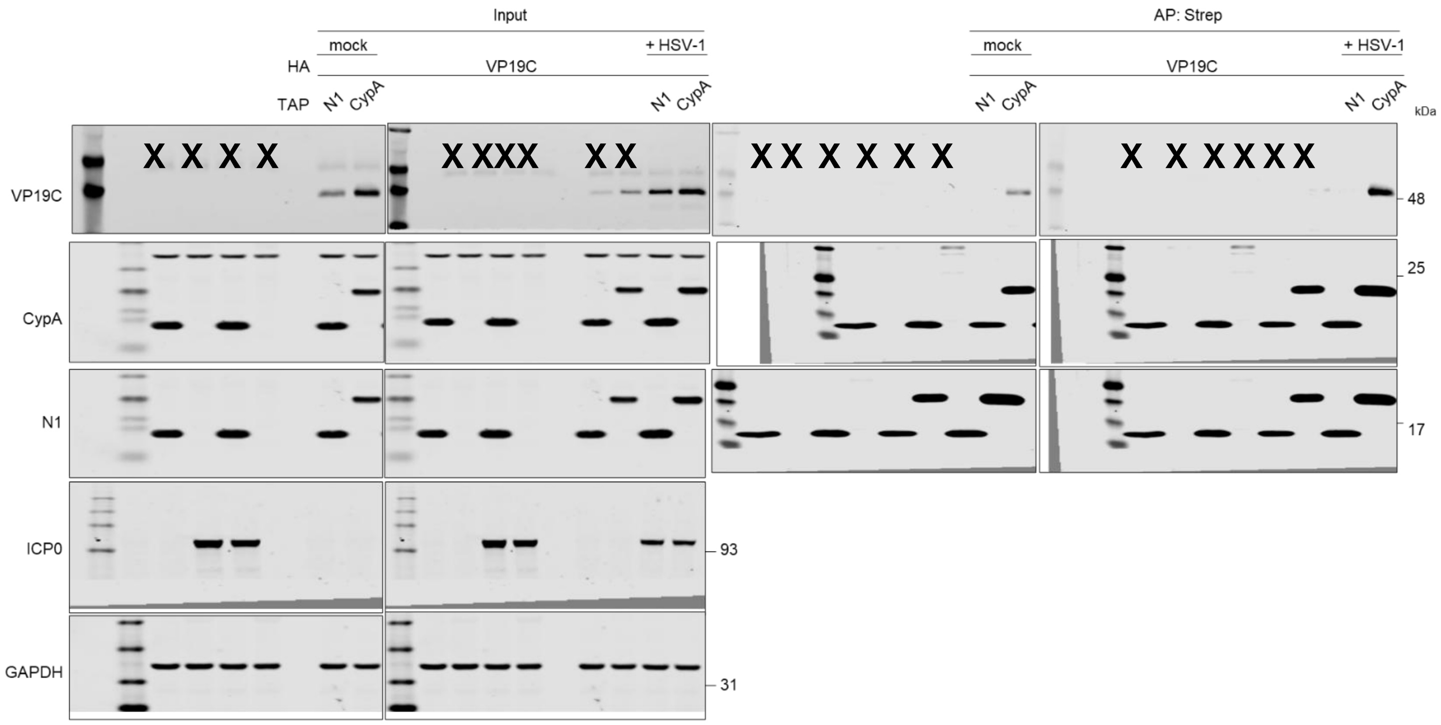
Input
AP: Strep
mock
+ HSV-1
HA
VP19C
TAP
N1
CypA
kDa
VP19C
CypA
N1
ICP0
GAPDH
48
25
17
93
31

Fig8b

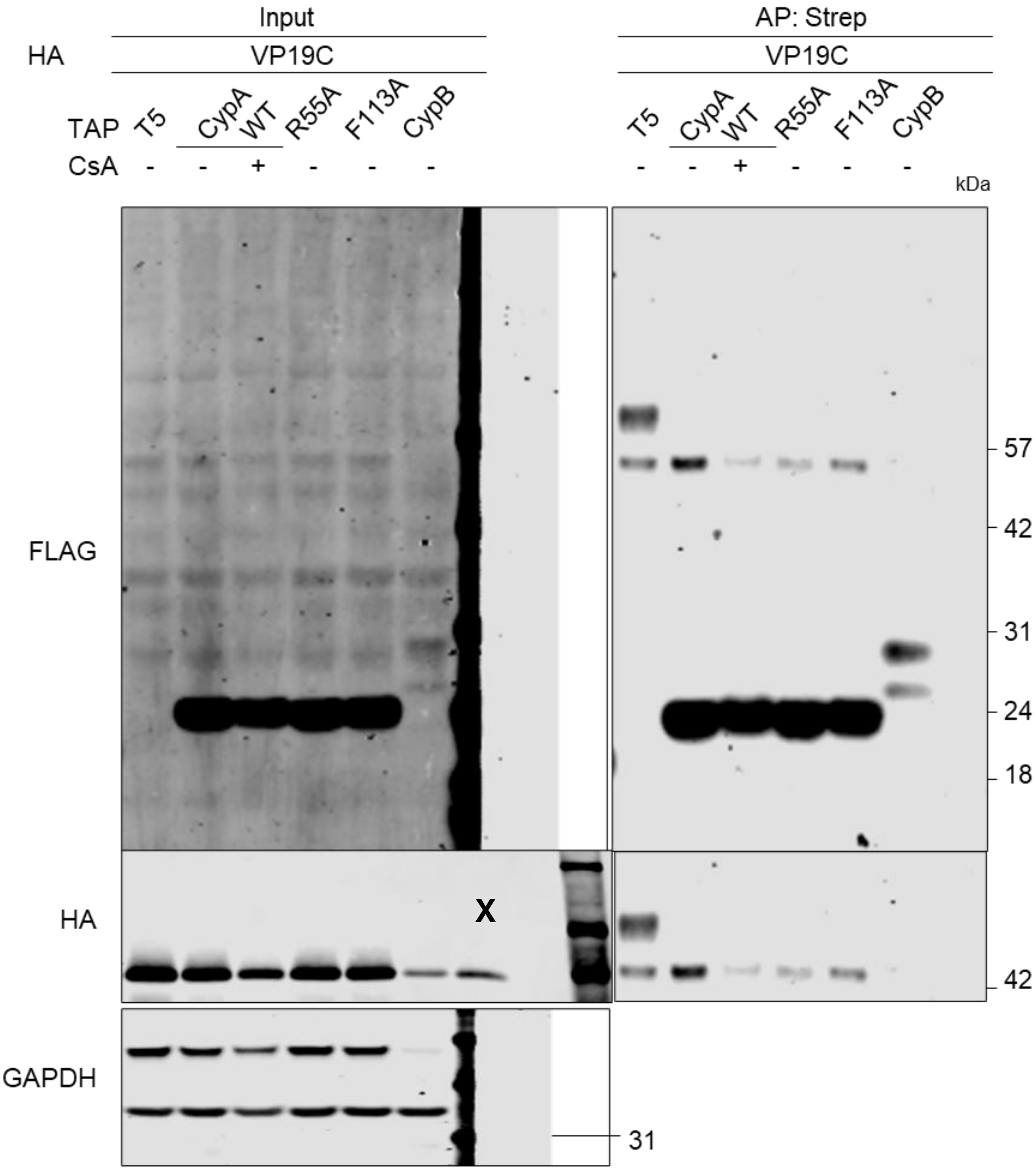

Input
AP: Strep
HA
VP19C
VP19C
TAP
T5
CypA
WT
R55A
F113A
CypB
CsA
-
+
kDa
FLAG
57
42
31
24
18
HA
X
42
GAPDH
31

Fig8c

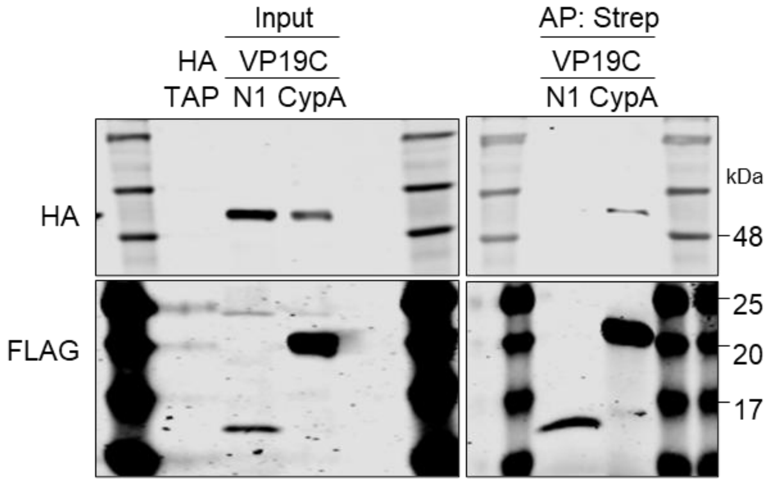
Input
HA
TAP
VP19C
N1 CypA
AP: Strep
VP19C
N1 CypA
HA
FLAG
kDa
48
25
20
17

Fig8d

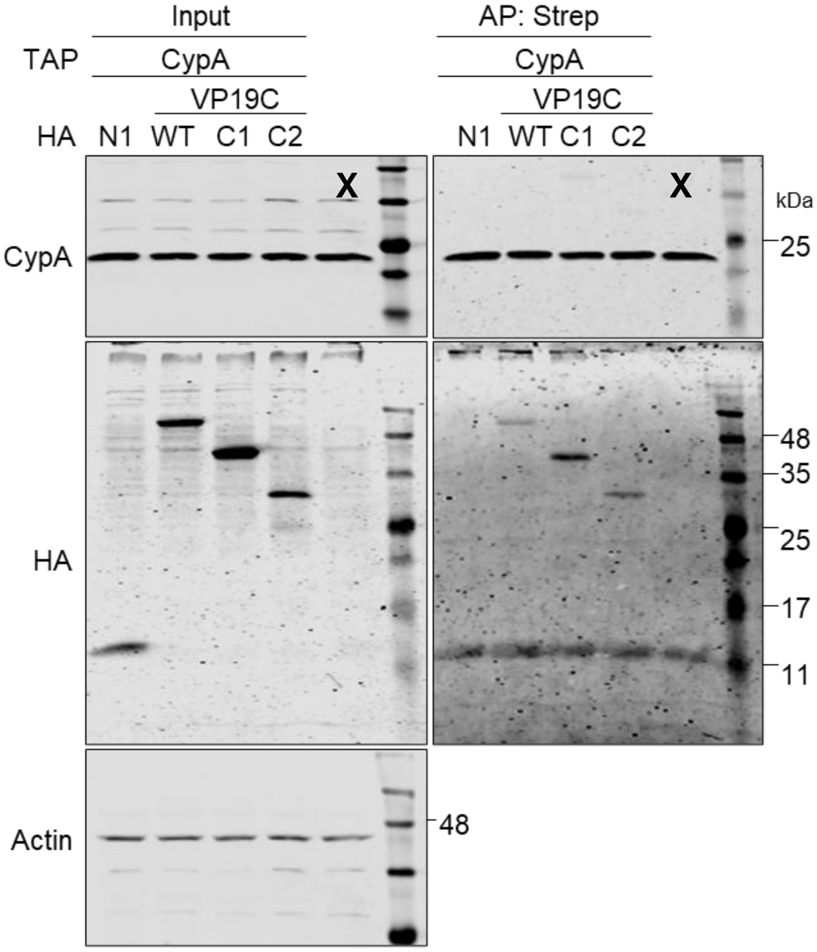

Input
AP: Strep
TAP
CypA
CypA
VP19C
VP19C
HA
N1
WT
C1
C2
N1
WT
C1
C2
X
X
kDa
25
CypA
48
35
25
17
11
HA
Actin
48

Fig8e

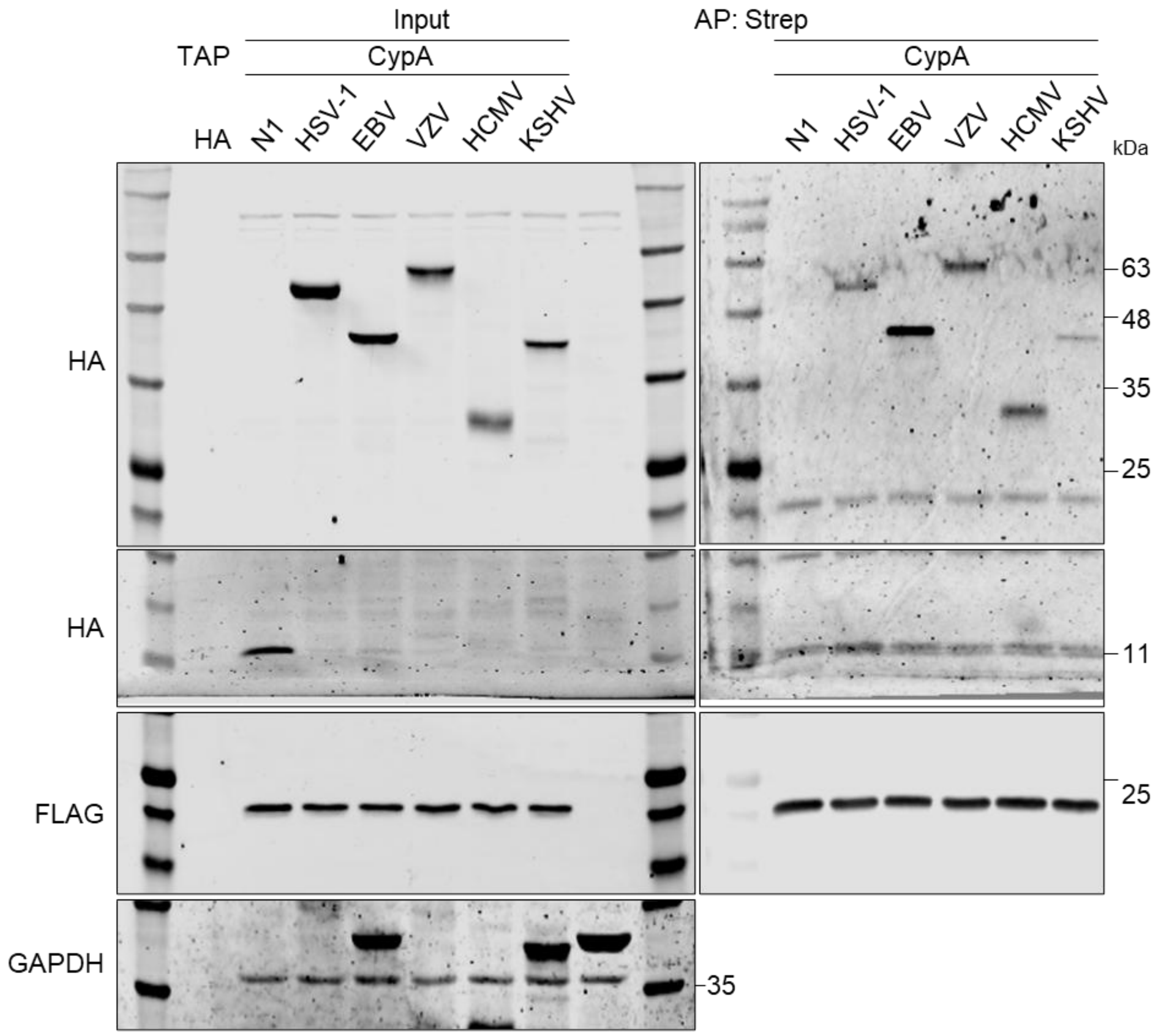
Input
AP: Strep
TAP
CypA
CypA
HA
N1
HSV-1
EBV
VZV
HCMV
KSHV
N1
HSV-1
EBV
VZV
HCMV
KSHV
kDa
HA
HA
FLAG
GAPDH
63
48
35
25
11
25
35

Fig8f

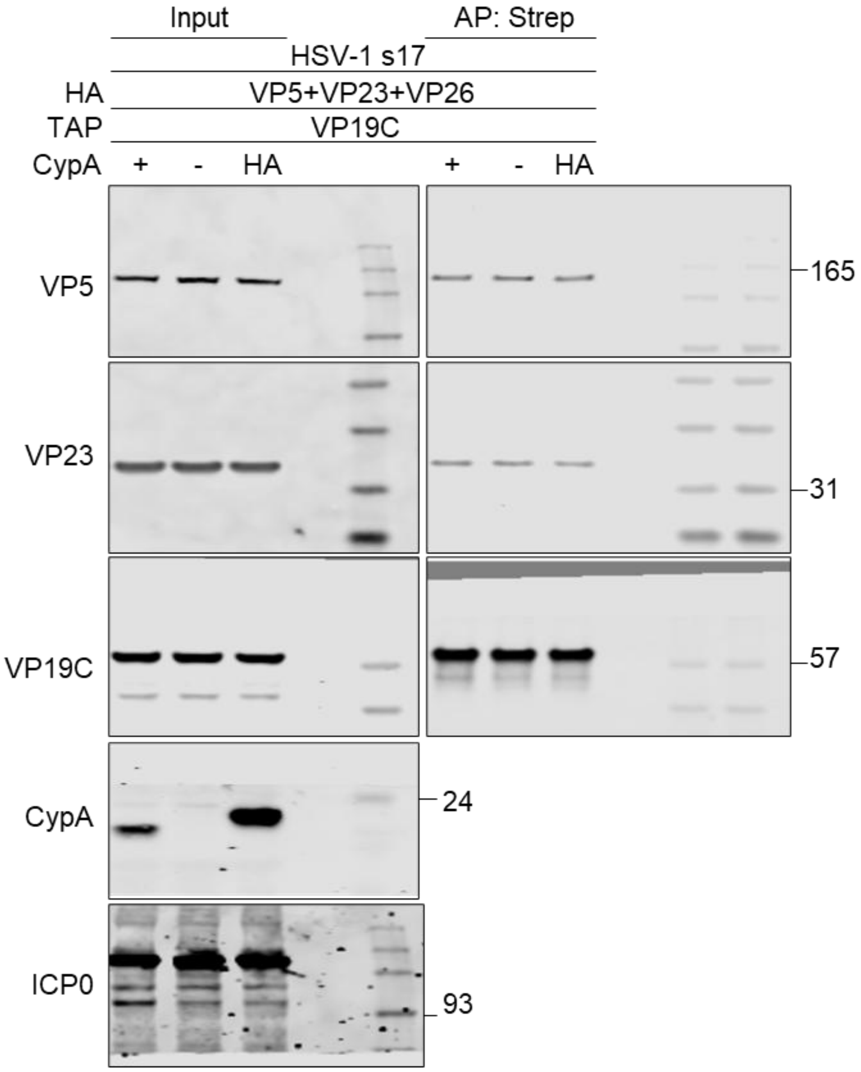

Input
AP: Strep
HSV-1 s17
HA
VP5+VP23+VP26
TAP
VP19C
CypA
+
-
HA
+
-
HA
VP5
165
VP23
31
VP19C
57
CypA
24
ICP0
93

Fig8g

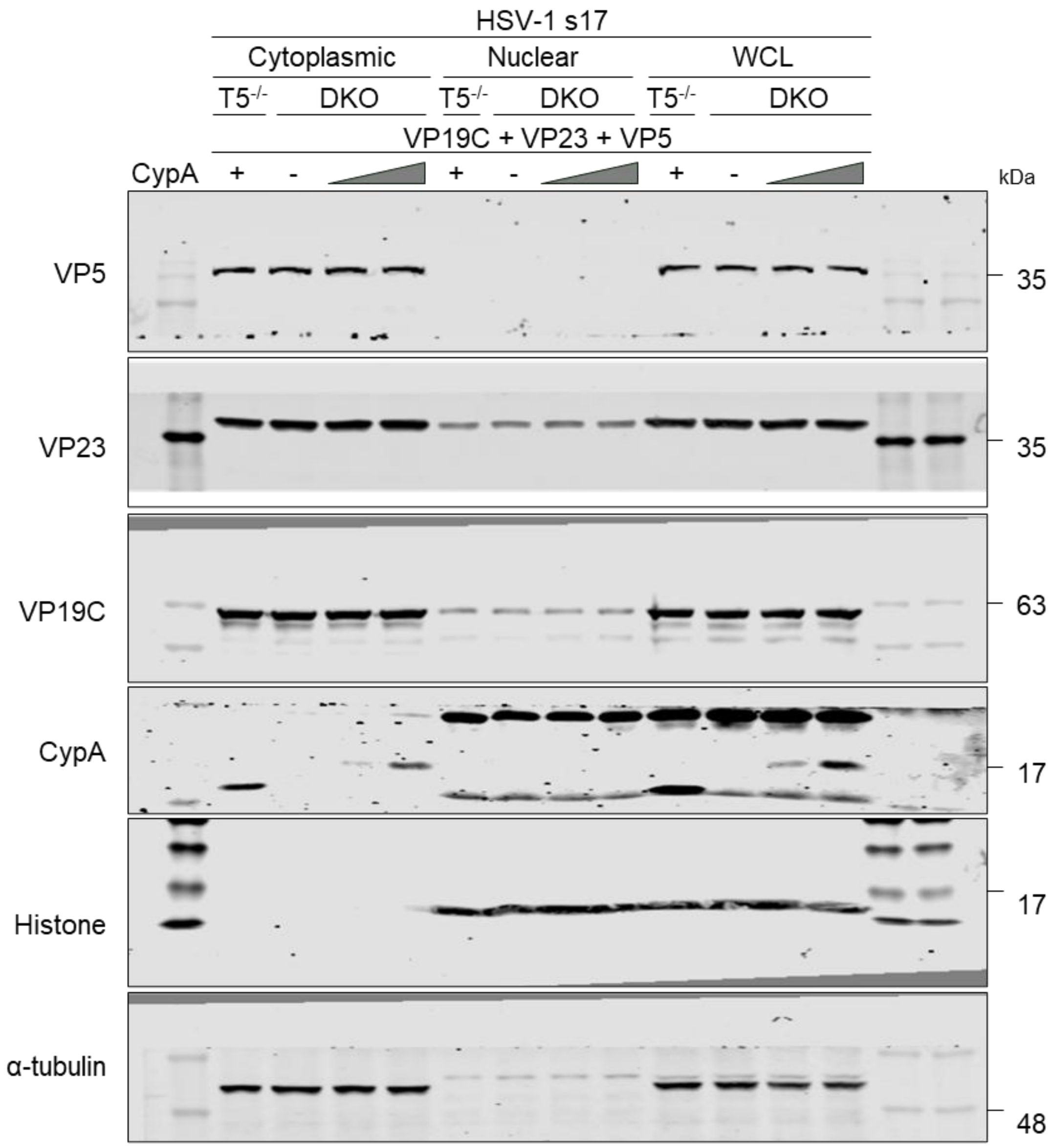
HSV-1 s17
Cytoplasmic
Nuclear
WCL
T5-/-
DKO
T5-/-
DKO
T5-/-
DKO
VP19C + VP23 + VP5
CypA
+
-
+
-
+
-
kDa
VP5
35
VP23
35
VP19C
63
CypA
17
Histone
17
α-tubulin
48
