# Supplementary material for: Independent proviral and antiviral host factors recognize the same capsid protein in divergent human herpesviruses
Source: PLoS Pathog. 2026 Jul 8;22(7):e1014376. doi: 10.1371/journal.ppat.1014376 (PMC13345423; doi:10.1371/journal.ppat.1014376)
Supplement: Attachment — Submitted filename: Response to Reviewers.docx [file ppat.1014376.s004.docx]

PPATHOGENS-D-26-00245
Independent proviral and antiviral host factors recognize the same capsid protein in divergent human herpesviruses.

Dear Editor

Thank you for your message about the above paper and for forwarding helpful comments from the reviewers. We are pleased that the paper was of interest and are able to respond to the individual comments are follows.

**Reviewer #1:**

1. The story here begins with the observation that HSV1 infection causes TRIM5 degradation in a proteasome-dependent manner. Can the authors explain how HSV1 degrades TRIM5? For example, does HSV-1 ICP0 degrade TRIM5? If such experiments were considered/attempted and there are technical issues with such experiments this might be mentioned.

We thank the reviewer for the suggestion to investigate ICP0. We had mentioned in the discussion section of the original submission (lines 351-9) that we had attempted to compare TRIM5 abundance during WT and ∆ICP0 HSV-1 infection, but were unable to do obtain equivalent infections because of the very poor replication of this mutant virus. As an alternative approach, we assessed TRIM5 degradation during ∆ICP0 HSV-1 infection in cell lines inducibly expressing ICP0 (+/- dox). We observed a slight rescue of TRIM5 degradation in the absence of ICP0 expression, but it was not to the same extent as the mock infection sample (figure below). Therefore, we could not confirm ICP0 as the viral protein responsible for TRIM5 degradation. We have not included these data in the results section of the current manuscript but could add it if deemed necessary.

Immunoblotting of TRIM5α abundance during ∆ICP0 HSV-1 infection in T-REx293 cells inducibly expressing ICP0 (+/- doxycycline) at 5 pfu/cell and 16 hpi. Data shown is representative of three independent experiments.

2. The authors show that the TRIM5 L19R mutant lacks restriction activity whereas the TRIM5 N70A mutant is like wild-type TRIM5. This suggests that synthesis of free K63-linked ubiquitin chains is required for anti-HSV activity as has been reported in the case of TRIM5 restriction of retroviruses (Nature 472:361). The latter work showed that association with the retroviral capsid lattice activates TRIM5 to synthesize free K63-linked ubiquitin chains, and that these K63-linked ubiquitin chains multimerize and activate TAK1 (MAP3K7) via its ubiquitin-binding cofactors, TAB2/3. Does genetic or pharmacologic disruption of TAK1 prevent TRIM5 restriction of HSV-1?

We thank the reviewer for the helpful suggestion to use a TAK1 inhibitor to dissect TRIM5-mediated restriction. We have now done this experiment and found that the inhibition of TAK1 enhanced virus replication and/or spread in the presence of TRIM5, but less so in its absence, showing that TRIM5 activates NF-kB as part of its anti-viral activity. Interestingly, infection in cells expressing TRIM5 yielded lower HSV-1 titres in the presence of the inhibitor compared to the knockout cells, suggesting that additional to innate signalling activation, there may be a second mechanism of restriction by TRIM5. We have included these data in Fig. 5f & g of the revised manuscript and described them on lines 241-7 of the results section.

**Reviewer #2:**

The authors present data on both TRIM5 and CypA, proposing that each binds to a minor capsid protein VP19C to alter HSV-1 infection. The phenotypes are quite weak for viral cofactors or restriction factors and consequently it is not convincing that either are crucial proteins in HSV-1 replication. The data supporting direct interaction relies heavily on pulldown experiments and would need to be supported by other more direct techniques. Overall the data feels superficial. My recommendation would be to divide up the initial findings presented here around CypA and TRIM5 and explore each in detail.

TRIM5 is naturally rapidly recycled via proteasomal degradation (PMID: 16472833), so how can HSV-1 promote something that is already happening very quickly?

We point out that the paper cited (PMID: 16472833) measured the turnover of over-expressed TRIM5 rather than endogenous TRIM5, and these might differ. It is also possible that TRIM5 stability might vary in different cell types.

But it is certainly possible for a virus to accelerate the degradation of a cellular protein such as TRIM5a. This has been demonstrated clearly with orthopoxviruses where a single virus protein called C6 (encoded by orthopoxvirus gene 29) can induce TRIM5a degradation either during infection, or in uninfected cells without any other virus protein (see Zhao et al., Nature 2023 PMID 37558876). So we are unsure why the reviewer thinks that this is not possible for HSV-1.

Its also worth noting that TRIM5 is an interferon stimulated gene (ISG) and its abundance is enhanced in response to interferon produced during infection. So there is definitely an advantage for the invading pathogen to induce its degradation and thereby antagonise TRIM5-mediated restriction.

Line 130. Figure 1a. Please remove as there no need to present previously published data. Or provide a repeat with replicate data.

We prefer to retain Fig. 1a, because, although the primary data were derived from a previous report, as we acknowledged, these data are part of a large dataset of thousands of cellular proteins and the stability of TRIM5a was not presented graphically or commented on. In our previous paper studying the stability of TRIM5a in vaccinia virus (VACV)-infected cells, the same situation existed, and in that case the editor and all reviewers approved graphical presentation of the stability of TRIM5a determined previously by mass spectrometry to lie alongside the new data we obtained by immunoblotting. We feel that presenting data from mass spectrometry and immunoblotting side by side strengthens the paper.

Line 132. Figure 1b. why are there multiple bands in the blot? what are they? Please show more of the blot and indicate more of the size markers so that the size of all the bands can be assessed. I am concerned that the antibody is not specific. Please show what the blot looks like in T5 negative cells.

We thank the reviewer for pointing out this potential confusion. We are confident that the antibody is specific because it was used to validate the TRIM5 knockout cell lines used in this study. Those cell lines were generated in our previous study (PMID: 37558876) and are referenced in the text. We have included an arrow to point out TRIM5 in the panel. Below is another immunoblot showing TRIM5 abundance in WT and TRIM5^-/-^ cells using the same antibody.

Immunoblotting showing TRIM5 abundance in WT and TRIM5^-/-^ T-REx293 cells.

Line 136. Figure 1d. N70A is a B-box mutant; it is still catalytically active. A mutant like L19R should be used here (as elsewhere in the manuscript).

N70A is an E3 ligase mutant that is defective in anchored polyubiquitin chain. This mutant was identified in a previous study (referenced in manuscript, PMID: 30503508) and shown to have an impaired turnover. We chose to use this mutant as it is expressed to higher levels than L19R so its abundance and degradation is clearer to see.

Line 150 Figure 2d. why do the negative cells display such substantially different viral titres? eg T5alpha- vs T5delta-. It suggests that the variation between the control cell lines is considerably greater than any specific T5-mediated effect. Same question for Figures 2e-h.

These cell lines have undergone single cell selection and so the variation is likely due to clonal differences. It is because of this that we engineered the cells to inducibly express protein of interest so that a better and more accurate comparison between +/- doxycycline (inducer) could be made.

Line 164. The data don't support such a specific claim. This would require data for each of the mutants showing that the formation of free chains (of what kind?) has been reduced.

The inability of L19R and ability of N70A to synthesise free polyubiquitin chains have been well characterised by others (Fig 1c, 1d in PMID: 30503508) and, previously, we and others have used this collection of TRIM5a mutants to determine which activities or domains are needed for anti-viral activity against retroviruses and poxviruses. The same mutants are used here to study TRIM5a-mediated restriction of HSV-1 and we believe the statement made is supported by the data presented.

Line 173. Figure 3a. Define N1 and explain why it is being used (I assume it's a control, but it is necessary to properly explain this).

The reviewer is correct, protein N1 is being used as negative control. Protein N1 is from vaccinia virus (VACV, encoded by orthopoxvirus gene 35) and is a Bcl-2-like protein. We have modified the text to explain its use and referenced its source.

Line 185. Figure 3d. Why does the WT full-length pulldown more weakly than C1?

Full-length VP19c has an N-terminal unstructured region, which does not bind to TRIM5 and might partially mask TRIM5’s binding site at the C-terminal region of VP19c. Therefore, removing this N-terminal unstructured region (e.g., the N-terminal truncation mutant C1) could improve the interaction between VP19c and TRIM5.

Line 190. Figure 3e. Is VP19C pull-down T5 dependent? Need AP:Strep negative control from cells not expressing T5.

The experiment shown in Fig. 3e was based on the validated VP19C-TRIM5 interaction. It shows that TAP-tagged-TRIM5 (bait) co-precipitates with HA-tagged capsid proteins (prey) from different herpesviruses, but not HA-tagged VACV protein N1. The cells used in this experiment are TRIM5^-/-^ T-REX293 so they do not express endogenous TRIM5.

Line 199. Figure 4. The resolution and magnification aren't sufficient to convincingly show T5 recruitment to the nuclear membrane. It seems only a subset of T5 positive cells are also positive for VP19C. Perhaps the authors could perform a quantitative comparison to measure the proportion of T5 at the nuclear membrane in control vs VP19C+ cells.

To address this point, we have included zoomed images. The cells used here are cell lines inducibly expressing TRIM5 (so all are TAP-tagged TRIM5 positive), transfected with epitope-tagged VP19C (and so only a subset of them are epitope-tagged VP19C positive) and infected with HSV-1. Due to the HSV-1 infection, the quantitative comparison in control vs VP19C+ cells cannot be performed because all of the cells are expressing VP19C, whether virus-encoded or epitope-tagged.

Line 206. Figure 5a. How does the data show that VP19C binding to VP23 is enhanced in presence of T5? It looks like T5 expression simply leads to more VP23 pulldown.

In this experiment, VP19C, VP23 and VP5 were co-expressed in the presence or absence of endogenous/epitope-tagged TRIM5. VP19C was used as bait and the other viral proteins as preys. The reviewer is correct in stating that both endogenous and HA-tagged TRIM5 expression leads to more VP23 pulldown where VP19C is the bait, and this is what we have concluded.

Line 208. Figure 5b. Please could you quantify the data as in 5a with replicates.

We have done this and added the quantification to the manuscript.

Line 229. Figure 5e. Is this activation of NFkB meaningful? Protein expression can nonspecifically activate NFkB. Further experiments are needed to understand if NFkB activation is happening during infection, whether this is sufficient to restrict virus and specifically dissect the contributions of VP19C and T5 to this induction.

To control for protein expression causing activation of NF-kB we included vaccinia virus proteins L3 and C1 as positive and negative controls respectively in the right 2 lanes as proteins that do or do not activate NF-kB. NF-kB activation by VP19c was then compared to these positive and negative controls.

We did consider testing NF-kB activation during HSV-1 infection when designing this experiment. However, because multiple HSV-1 proteins inhibit NF-kB activation, fold activation during infection will be diminished by these (as well as by the virus-induced TRIM5 degradation) (PMID: [24067962](https://pubmed.ncbi.nlm.nih.gov/24067962/), PMID: [24807716](https://pubmed.ncbi.nlm.nih.gov/24807716/), PMID: [32477319](https://pubmed.ncbi.nlm.nih.gov/32477319/), PMID: [28100608](https://pubmed.ncbi.nlm.nih.gov/28100608/), PMID: [28320502](https://pubmed.ncbi.nlm.nih.gov/28320502/), PMID: [28031360](https://pubmed.ncbi.nlm.nih.gov/28031360/)), and as a result, VP19C- and TRIM5-induced activation might be masked. Therefore, to be able to better understand how TRIM5 and VP19C contribute to NF-kB activation, we have performed the experiment outwith infection.

Line 234. Figure 6a. < 2-fold difference is not meaningful when studying viral replication.

We agree that the effect of TRIM5 KO in T-REx-293 is less than two-fold for one of the KO cell line after high MOI, however the difference is still statistically significant. In the low MOI experiments, the effect of T5 KO is more evident, where reduction is around 2-fold and the virus plaque sizes are visibly larger so, respectfully, we diasagree with this comment.

We feel it is important to bear in mind that TRIM5-mediated restriction shown in these titration and plaque size studies is a net result of TRIM5 antiviral activity vs virus-induced TRIM5 degradation (and other unknown evasion strategies). So the magnitude of TRIM5 restriction will be greater in the absence of the viral protein responsible for its down-regulation. Furthermore, virus-host interaction is a subtle balance of host defense and viral evasion so we cannot expect the restriction exerted by a single host protein to dictate infection outcome, rather, it is a synergistic effect of many cellular restriction factors.

Line 246. Figure 6b. Are these technical replicates or from independent biological experiments using different viral preps and done on different days. The lack of variability between repeats, eg F113A + Dox, is remarkable even for technical replicates.

These are independent replicates within a single experiment and using the same virus stock. This experiment has been repeated three times and each time, the data showed the same phenotype +/- dox for each of the cell lines.

Line 250. Figure 6d. The phenotype for CsA is more substantial than ± CypA, suggesting the compound has other affects beyond CypA. To test this the authors should perform ± CypA at 20 µM in the panel of cells shown in 6b to see if there is CypA-independent effects.

As suggested, we have done this experiment and found that other PPIs also play roles during HSV-1 infection. We are currently investigating this in other ongoing projects and feel that including these data here is straying from the focus of this study.

We hope these additional data and explanations are satisfactory and look forward to your response in due course.

Yours sincerely,

Yiqi ZHAO

Yongxu LU

Geoffrey L SMITH
