# Supplementary material for: Independent proviral and antiviral host factors recognize the same capsid protein in divergent human herpesviruses
Source: PLoS Pathog. 2026 Jul 8;22(7):e1014376. doi: 10.1371/journal.ppat.1014376 (PMC13345423; doi:10.1371/journal.ppat.1014376)
Supplement: Attachment — Submitted filename: Response_to_Reviewers_auresp_2.docx [file ppat.1014376.s005.docx]

PPATHOGENS-D-26-00245R1
Independent proviral and antiviral host factors recognize the same capsid protein in divergent human herpesviruses
PLOS Pathogens

Dear Editor

Thank you for your message about the above paper and for forwarding helpful comments from the reviewers. We are pleased that the paper was of interest and are able to respond to the individual comments are follows.

**Additional Editor Comments (if provided):**

One of the original reviewers was unable to provide a re-review, so a third reviewer was recruited to comment on whether the original reviews had been addressed. They have confirmed that the original review questions have been met, but raise two additional points - one relates to an immunofluorescence figure and the other to a point for the discussion. These questions are important but fair - the latter can be addressed through text changes, the former may also be explainable through text changes or experimentation.

We thank the editor for these summary comments. Our comments are given below.

**Journal Requirements:**

If the reviewer comments include a recommendation to cite specific previously published works, please review and evaluate these publications to determine whether they are relevant and should be cited. There is no requirement to cite these works unless the editor has indicated otherwise.

1) We have noticed that you have uploaded Supporting Information files, but you have not included a list of legends. Please add a full list of legends for your Supporting Information files after the references list.

As requested a list of legends for the supporting information files is added after the references list.

2) PLOS now requires that authors provide the original uncropped and unadjusted images underlying all blot or gel results reported in a submission’s figures or Supporting Information files. This policy and the journal’s other requirements for blot/gel reporting and figure preparation are described in detail at https://journals.plos.org/plosone/s/figures#loc-blot-and-gel-reporting-requirements and https://journals.plos.org/plosone/s/figures#loc-preparing-figures-from-image-files.

When you submit your revised manuscript, please ensure that your figures adhere fully to these guidelines and provide the original underlying images for all blot or gel data reported in your submission. See the following link for instructions on providing the original image data: https://journals.plos.org/plosone/s/figures#loc-original-images-for-blots-and-gels.

In your cover letter, please note whether your blot/gel image data are in Supporting Information or posted at a public data repository, provide the repository URL if relevant, and provide specific details as to which raw blot/gel images, if any, are not available. Email us at [customercare@plos.org](mailto:customercare@plos.org) if you have any questions.

We have uploaded the raw image file to the submission portal and have deposited at <https://figshare.com/s/71963eaa018030777759> with an embargo until publication.

**Reviewers' Comments:**

Reviewer's Responses to Questions

**Part I - Summary**
Please use this section to discuss strengths/weaknesses of study, novelty/significance, general execution and scholarship.

Reviewer #1: The authors have addressed my previous suggestions.

Reviewer #3: Zhao et al. submitted a revised manuscript reporting novel results showing interactions of the host proteins TRIM5alpha and cyclophilin A with the VP19c subunit of the capsid triplex of herpes simplex virus (HSV) and its orthologs in VZV, HCMV, EBV, and KSHV.

The authors furthermore show, for the first time, that the interferon-inducible TRIM5alpha is a restriction factor for HSV-1, and that this activity requires its Ub ligase activity, the PRY-SPRY domain, and oligomerization. Moreover, they report that VP19c induces NF-kappaB signalling independently and synergistically with TRIM5alpha. In contrast, the other host factor, cyclophilin A, is proviral; however, for HSV-1, unlike other viral infections, this proviral activity does not depend on TRIM5alpha. Based on these data, the authors propose testing whether cyclosporin A, which targets cyclophilin and inhibits neuronal HSV infections, could be useful in treating HSV encephalitis.

A major weakness of this study is that the authors did not address whether TRIM5alpha and cyclophilin A interact only with soluble forms of VP19c and its orthologs, or whether they also interact with VP19c after its incorporation into viral particles. The authors did not conduct any experiments with viral capsids, yet they frame their study in the context of viral capsids as potential targets for pattern recognition receptors (introduction, lines 75-76; Results, lines 196; discussion, lines 315 - 319). On the other hand, the authors missed that the GTPase Mx2 (MxB) has already been shown to limit HIV and HSV infection by restricting viral capsids.

We regret omitting reference to the work on Mx2 limiting HIV and HSV replication, and are grateful for the opportunity to do so now. This is now mentioned on lines 361-362 of the revised paper.

We agree that experiments to address whether TRIM5a or CypA interact with vP19C in capsids are important, but they are not straightforward and to conduct this thoroughly is a significant undertaking. For instance, CypA has been found within HSV-1 capsids already (PMID: [18596102](https://pubmed.ncbi.nlm.nih.gov/18596102/)), but to prove if this is via an interaction with VP19C it would be necessary to do experiments with capsids that do or not make VP19C and this is not easy because of the important role of VP19C in virus replication. An alternative approach of using a virus with a mutant VP19C that retains every known function except binding TRIM5a or CypA is not possible given the lack of precise information about how these proteins interact. We feel that this question for both TRIM5a and CypA is beyond the scope of this first paper on this new phenomenon.

Furthermore, figure 4 does not align with published studies by other groups on the subcellular localization of capsids and capsid proteins like VP19c during HSV-1 infection.

The reviewer has misunderstood Fig. 4 and this is because we did not write a full enough legend, for which we apologise. We have now corrected this in the figure legend. These cells express TAP-tagged TRIM5a and were also transfected with a tagged-VP19C before infection by HSV-1 at high MOI. The column of panels labelled VP19C show cells in which the transfected VP19C is detected by an antibody to the tag. This explains why: i) some cells are positive for VP19C in mock-infected cells, ii) not all cells express the tagged VP19C, and iii) the overexpression of the cell with VP19C has caused aberrant localization of VP19C (this was noted and optimisations to reduce this was attempted but not successful, therefore we did not make conclusions related to VP19C and TRIM5 co-localisation in the manuscript).

We had included tagged VP19C because we wanted to address whether the overexpression of VP19C affected TRIM5a distribution, and it is clear from the images that it did not (at least not when all cells are also expressing endogenous VP19C made during infection). But this was not the real point of Fig. 4. The real point concerns the distribution of TRIM5a before and during infection. Here we certainly disagree with reviewer 3 and we assert that there is a clear redistribution of TRIM5a to around the nuclear membrane as infection progresses. Compare the mock infected and 4 h time point with the later ones of 7 and 10 hpi. The change is very clear with or without the zoomed image. Furthermore, this re-distribution did not occur when a TRIM5a mutant lacking the SPRY domain (needed for interaction with VP19C) was expressed. So on this point we stand by our original statements.

**Part II – Major Issues: Key Experiments Required for Acceptance**
Please use this section to detail the key new experiments or modifications of existing experiments that should be absolutely required to validate study conclusions.

Generally, there should be no more than 3 such required experiments or major modifications for a "Major Revision" recommendation. If more than 3 experiments are necessary to validate the study conclusions, then you are encouraged to recommend "Reject".

Reviewer #1: N/A

Reviewer #3: Overall, the authors have adequately addressed the reviewer's comments on the first submission.

However, the data in Figure 4 is still confusing. It is only briefly described in the results section, and the legend provides little information.

Why is there a signal for VP19C in the mock-condition? See explanation above

Is Mock the 0 h time point? Mock was uninfected cells harvested at the last time point.

None of the images, including those in the zoom setting, indicate that TRIM5alpha is targeted to or enriched at the nuclear envelope. All images suggest a rather uniform distribution throughout the entire cytoplasm. We disagree with these 2 statements and assert that there is a clear redistribution of TRIM5a to the nuclear membrane. This occurs with WT TRIM5a but not the mutant TRIM5a tested, which is not antiviral.

Furthermore, the images are inconsistent with the concept that TRIMalpha5 restricts HSV-1 infection. Some cells show a strong VP19c signal, while other cells do not reveal any VP19c signal at all. Furthermore, VP19c is a capsid protein; thus, it should be efficiently targeted to the nucleus (as also described in the introduction), and its accumulation should increase over time in the nucleus and later in the cytoplasm. These data are inconsistent with other groups' reports on the subcellular localization of capsids and capsid proteins during an HSV-1 infection.

This is all explained above. The VP19C detected is tagged VP19C transfected into some cells and not others.

Furthermore, these data do not align with the authors' description in the discussion (lines 330-332). It is not true that there are no antibodies directed against HSV-1 capsid proteins and suitable for immunofluorescence microscopy (discussion, line 342). Furthermore, several HSV-1 strains are available in which the capsid proteins have been tagged with autofluorescent proteins, which could be used in cells expressing different versions of TRIMalpha5.

We had not stated that no antibodies to any HSV-1 capsid protein was available, but rather to the capsid proteins we were studying (“*we were restricted by the lack of antibodies against these herpesvirus capsid proteins.”)*. We have added a statement in the results (line 350) to make explicit which capsid proteins were are referring to. HSV-1 strains expressing autofluorescently-tagged proteins might be useful for co-localisation studies, but our experience with similarly labelled poxvirus capsids is that the strength of the signal from the autofluorescently-tagged viral capsid masks weak signals from cellular proteins that might be recruited to the incoming capsid. So again, these experiments are not straightforward and we feel they are outside the scope of this study.

**Part III – Minor Issues: Editorial and Data Presentation Modifications**
Please use this section for editorial suggestions as well as relatively minor modifications of existing data that would enhance clarity.

Reviewer #1: N/A

Reviewer #3: The authors should refer to Mx1 as an established interferon-inducible host protein that has been proven to restrict the functions of viral capsids, and address in their discussion the limitation of their present work as they have not elucidated whether TRIM5alpha limits HSV-1 infection by perturbing capsid functions or just intracellular trafficking of the capsid protein V19c. or by activating innate immunity, or by some combination of these.

We are pleased to include reference to the Mx proteins as suggested in the discussion (lines 361-362).

We hope these additional explanations are satisfactory and look forward to your response in due course.

Yours sincerely,

Yiqi ZHAO

Yongxu LU

Geoffrey L SMITH
