# Supplementary material for: Independent proviral and antiviral host factors recognize the same capsid protein in divergent human herpesviruses
Source: PLoS Pathog. 2026 Jul 8;22(7):e1014376. doi: 10.1371/journal.ppat.1014376 (PMC13345423; doi:10.1371/journal.ppat.1014376)
Supplement: Peer review history

# Peer review: Decision Letter 0

**DOI:** 10.1371/journal.ppat.1014376.r001

**Authors:** Richard James Stanton, Donna M Neumann

16 Mar 2026

-->PPATHOGENS-D-26-00245

Independent proviral and antiviral host factors recognize the same capsid protein in divergent human herpesviruses

PLOS Pathogens

Dear Dr. Lu,

Thank you for submitting your manuscript to PLOS Pathogens. After careful consideration, we feel that it has merit but does not fully meet PLOS Pathogens's publication criteria as it currently stands. Therefore, we invite you to submit a revised version of the manuscript that addresses the points raised during the review process.

Please submit your revised manuscript by May 15 2026 11:59PM. If you will need more time than this to complete your revisions, please reply to this message or contact the journal office at plospathogens@plos.org. When you're ready to submit your revision, log on to https://www.editorialmanager.com/ppathogens/ and select the 'Submissions Needing Revision' folder to locate your manuscript file.

Please include the following items when submitting your revised manuscript:

* A letter that responds to each point raised by the editor and reviewer(s). You should upload this letter as a separate file labeled 'Response to Reviewers'. This file does not need to include responses to any formatting updates and technical items listed in the 'Journal Requirements' section below.

* A marked-up copy of your manuscript that highlights changes made to the original version. You should upload this as a separate file labeled 'Revised Manuscript with Track Changes'.

* An unmarked version of your revised paper without tracked changes. You should upload this as a separate file labeled 'Manuscript'.

If you would like to make changes to your financial disclosure, competing interests statement, or data availability statement, please make these updates within the submission form at the time of resubmission. Guidelines for resubmitting your figure files are available below the reviewer comments at the end of this letter.

We look forward to receiving your revised manuscript.

Kind regards,

Richard James Stanton

Academic Editor

PLOS Pathogens

Donna Neumann

Section Editor

PLOS Pathogens

Sumita Bhaduri-McIntosh

Editor-in-Chief

PLOS Pathogens

orcid.org/0000-0003-2946-9497

Michael Malim

Editor-in-Chief

PLOS Pathogens

orcid.org/0000-0002-7699-2064

**Journal Requirements:**

1) Please ensure that the CRediT author contributions listed for every co-author are completed accurately and in full.

At this stage, the following Authors/Authors require contributions: Yiqi Zhao, Sarah Probert, Jan Birkel, Xin Liu, Yaling Shi, Olivia-Shulan Yang, Kangyan Zhao, Yongxu Lu, and Geoffrey Smith. Please ensure that the full contributions of each author are acknowledged in the "Add/Edit/Remove Authors" section of our submission form.

The list of CRediT author contributions may be found here: https://journals.plos.org/plospathogens/s/authorship#loc-author-contributions

2) We ask that a manuscript source file is provided at Revision. Please upload your manuscript file as a .doc, .docx, .rtf or .tex. If you are providing a .tex file, please upload it under the item type u2018LaTeX Source Fileu2019 and leave your .pdf version as the item type u2018Manuscriptu2019.

3) Please upload all main figures as separate Figure files in .tif or .eps format. For more information about how to convert and format your figure files please see our guidelines:

https://journals.plos.org/plospathogens/s/figures

4) We notice that your supplementary Tables are included in the manuscript file. Please remove them and upload them with the file type 'Supporting Information'. Please ensure that each Supporting Information file has a legend listed in the manuscript after the references list.

5) When completing the data availability statement of the submission form, you indicated that you will make your data available on acceptance. We strongly recommend all authors decide on a data sharing plan before acceptance, as the process can be lengthy and hold up publication timelines. Please note that, though access restrictions are acceptable now, your entire data will need to be made freely accessible if your manuscript is accepted for publication. This policy applies to all data except where public deposition would breach compliance with the protocol approved by your research ethics board. If you are unable to adhere to our open data policy, please kindly revise your statement to explain your reasoning and we will seek the editor's input on an exemption. Please be assured that, once you have provided your new statement, the assessment of your exemption will not hold up the peer review process.

6) Please amend your detailed Financial Disclosure statement. This is published with the article. It must therefore be completed in full sentences and contain the exact wording you wish to be published.

1) State the initials, alongside each funding source, of each author to receive each grant. For example: "This work was supported by the National Institutes of Health (####### to AM; ###### to CJ) and the National Science Foundation (###### to AM)."

2) State what role the funders took in the study. If the funders had no role in your study, please state: "The funders had no role in study design, data collection and analysis, decision to publish, or preparation of the manuscript.".

Note: If the reviewer comments include a recommendation to cite specific previously published works, please review and evaluate these publications to determine whether they are relevant and should be cited. There is no requirement to cite these works unless the editor has indicated otherwise.

**Reviewers' Comments:**

Reviewer's Responses to Questions

**Part I - Summary**

Please use this section to discuss strengths/weaknesses of study, novelty/significance, general execution and scholarship.

Reviewer #1: The authors used mass spec to screen for changes in host proteins due to HSV-1 infection. It was found that TRIM5 protein levels were reduced, MG132 prevented the reduction, and TRIM5 KO increased viral titer and plaque size. Complementation of the knockout cells with various TRIM5 isoforms and mutants showed that the anti-HSV-1 activity required the alpha isoform, oligomerization of the C-terminal PRY-SPRY domain, and the production of unanchored polyubiquitin chains. Based on the TRIM5 virus literature (retroviruses and EBV), the authors found that TRIM5 binds to HSV VP19C and to homologs from other herpes viruses. TRIM5 promoted VP19C interaction with VP23 but not VP5, and nuclear accumulation of both VP19C and VP23. VP19C overexpression activated an NFkB reporter in a TRIM5-dependent manner. Finally, since cyclophilin A modulates TRIM5 restriction activity of HIV-1, the authors checked the effect of cyclophilin A: the titer of HSV-1 was reduced by cyclophilin knockout or by drugs that block cyclophilin. Cyclophilin also binds to VP19C and the homologous proteins in the other viruses but the effects of CypA on HSV-1 were independent of TRIM5.

This manuscript reports important findings concerning restriction of HSV-1 by the capsid-specific innate receptor TRIM5, as well as positive effects on HSV-1 replication of the capsid-binding protein cyclophilin A. The data are convincing, clearly presented, and biologically meaningful. The manuscript extends the scope of virus capsid detection by TRIM5 and cyclophilin A to herpes viruses, an important contribution to the literature.

Reviewer #2: The authors present data on both TRIM5 and CypA, proposing that each binds to a minor capsid protein VP19C to alter HSV-1 infection. The phenotypes are quite weak for viral cofactors or restriction factors and consequently it is not convincing that either are crucial proteins in HSV-1 replication. The data supporting direct interaction relies heavily on pulldown experiments and would need to be supported by other more direct techniques. Overall the data feels superficial. My recommendation would be to divide up the initial findings presented here around CypA and TRIM5 and explore each in detail.

TRIM5 is naturally rapidly recyled via proteasomal degradation (PMID: 16472833), so how can HSV-1 promote something that is already happening very quickly?

Line 130. Figure 1a. Please remove as there no need to present previously published data. Or provide a repeat with replicate data.

Line 132. Figure 1b. why are there multiple bands in the blot? what are they? Please show more of the blot and indicate more of the size markers so that the size of all the bands can be assessed. I am concerned that the antibody is not specific. Please show what the blot looks like in T5 negative cells.

Line 136. Figure 1d. N70A is a B-box mutant; it is still catalytically active. A mutant like L19R should be used here (as elsewhere in the manuscript).

Line 150 Figure 2d. why do the negative cells display such substantially different viral titres? eg T5alpha- vs T5delta-. It suggests that the variation between the control cell lines is considerably greater than any specific T5-mediated effect. Same question for Figures 2e-h.

Line 164. The data don't support such a specific claim. This would require data for each of the mutants showing that the formation of free chains (of what kind?) has been reduced.

Line 173. Figure 3a. Define N1 and explain why it is being used (I assume it's a control, but it is necessary to properly explain this).

Line 185. Figure 3d. Why does the WT full-length pulldown more weakly than C1?

Line 190. Figure 3e. Is VP19C pull-down T5 dependent? Need AP:Strep negative control from cells not expressing T5.

Line 199. Figure 4. The resolution and magnification aren't sufficient to convincingly show T5 recruitment to the nuclear membrane. It seems only a subset of T5 positive cells are also positive for VP19C. Perhaps the authors could perform a quantitative comparison to measure the proportion of T5 at the nuclear membrane in control vs VP19C+ cells.

Line 206. Figure 5a. How does the data show that VP19C binding to VP23 is enhanced in presence of T5? It looks like T5 expression simply leads to more VP23 pulldown.

Line 208. Figure 5b. Please could you quantify the data as in 5a with replicates.

Line 229. Figure 5e. Is this activation of NFkB meaningful? Protein expression can nonspecifically activate NFkB. Further experiments are needed to understand if NFkB activation is happening during infection, whether this is sufficient to restrict virus and specifically dissect the contributions of VP19C and T5 to this induction.

Line 234. Figure 6a. < 2-fold difference is not meaningful when studying viral replication.

Line 246. Figure 6b. Are these technical replicates or from independent biological experiments using different viral preps and done on different days. The lack of variability between repeats, eg F113A + Dox, is remarkable even for technical replicates.

Line 250. Figure 6d. The phenotype for CsA is more substantial than ± CypA, suggesting the compound has other affects beyond CypA. To test this the authors should perform ± CypA at 20 µM in the panel of cells shown in 6b to see if there is CypA-independent effects.

**********

**Part II – Major Issues: Key Experiments Required for Acceptance**

Please use this section to detail the key new experiments or modifications of existing experiments that should be absolutely required to validate study conclusions.

Generally, there should be no more than 3 such required experiments or major modifications for a "Major Revision" recommendation. If more than 3 experiments are necessary to validate the study conclusions, then you are encouraged to recommend "Reject".

Reviewer #1: (No Response)

Reviewer #2: (No Response)

**********

**Part III – Minor Issues: Editorial and Data Presentation Modifications**

Please use this section for editorial suggestions as well as relatively minor modifications of existing data that would enhance clarity.

Reviewer #1: 1. The story here begins with the observation that HSV1 infection causes TRIM5 degradation in a proteasome-dependent manner. Can the authors explain how HSV1 degrades TRIM5? For example, does HSV-1 ICP0 degrade TRIM5? If such experiments were considered/attempted and there are technical issues with such experiments this might be mentioned.

2. The authors show that the TRIM5 L19R mutant lacks restriction activity whereas the TRIM5 N70A mutant is like wild-type TRIM5. This suggests that synthesis of free K63-linked ubiquitin chains is required for anti-HSV activity as has been reported in the case of TRIM5 restriction of retroviruses (Nature 472:361). The latter work showed that association with the retroviral capsid lattice activates TRIM5 to synthesize free K63-linked ubiquitin chains, and that these K63-linked ubiquitin chains multimerize and activate TAK1 (MAP3K7) via its ubiquitin-binding cofactors, TAB2/3. Does genetic or pharmacologic disruption of TAK1 prevent TRIM5 restriction of HSV-1?

Reviewer #2: (No Response)

**********

PLOS authors have the option to publish the peer review history of their article (what does this mean?). If published, this will include your full peer review and any attached files.

If you choose “no”, your identity will remain anonymous but your review may still be made public.

**Do you want your identity to be public for this peer review?**  For information about this choice, including consent withdrawal, please see our Privacy Policy.

Reviewer #1: No

Reviewer #2: No

[NOTE: If reviewer comments were submitted as an attachment file, they will be attached to this email and accessible via the submission site. Please log into your account, locate the manuscript record, and check for the action link "View Attachments". If this link does not appear, there are no attachment files.]

**Figure resubmission:**

-->While revising your submission, we strongly recommend that you use PLOS’s NAAS tool (https://ngplosjournals.pagemajik.ai/artanalysis) to test your figure files. NAAS can convert your figure files to the TIFF file type and meet basic requirements (such as print size, resolution), or provide you with a report on issues that do not meet our requirements and that NAAS cannot fix.

After uploading your figures to PLOS’s NAAS tool - https://ngplosjournals.pagemajik.ai/artanalysis, NAAS will process the files provided and display the results in the "Uploaded Files" section of the page as the processing is complete. If the uploaded figures meet our requirements (or NAAS is able to fix the files to meet our requirements), the figure will be marked as "fixed" above. If NAAS is unable to fix the files, a red "failed" label will appear above. When NAAS has confirmed that the figure files meet our requirements, please download the file via the download option, and include these NAAS processed figure files when submitting your revised manuscript.

**Reproducibility:**

To enhance the reproducibility of your results, we recommend that authors of applicable studies deposit laboratory protocols in protocols.io, where a protocol can be assigned its own identifier (DOI) such that it can be cited independently in the future. Additionally, PLOS ONE offers an option to publish peer-reviewed clinical study protocols. Read more information on sharing protocols at https://plos.org/protocols?utm_medium=editorial-email&utm_source=authorletters&utm_campaign=protocols

# Author comment: Author response to Decision Letter 1

**DOI:** 10.1371/journal.ppat.1014376.r002

11 May 2026

Attachment Submitted filename: Response to Reviewers.docx

# Peer review: Decision Letter 1

**DOI:** 10.1371/journal.ppat.1014376.r003

**Authors:** Richard James Stanton, Donna M Neumann

10 Jun 2026

PPATHOGENS-D-26-00245R1

Independent proviral and antiviral host factors recognize the same capsid protein in divergent human herpesviruses

PLOS Pathogens

Dear Dr. Zhao,

Thank you for submitting your manuscript to PLOS Pathogens. After careful consideration, we feel that it has merit but does not fully meet PLOS Pathogens's publication criteria as it currently stands. Therefore, we invite you to submit a revised version of the manuscript that addresses the points raised during the review process.

Please submit your revised manuscript by Aug 09 2026 11:59PM. If you will need more time than this to complete your revisions, please reply to this message or contact the journal office at plospathogens@plos.org. When you're ready to submit your revision, log on to https://www.editorialmanager.com/ppathogens/ and select the 'Submissions Needing Revision' folder to locate your manuscript file.

Please include the following items when submitting your revised manuscript:

* A letter that responds to each point raised by the editor and reviewer(s). You should upload this letter as a separate file labeled 'Response to Reviewers'. This file does not need to include responses to any formatting updates and technical items listed in the 'Journal Requirements' section below.

* A marked-up copy of your manuscript that highlights changes made to the original version. You should upload this as a separate file labeled 'Revised Manuscript with Track Changes'.

* An unmarked version of your revised paper without tracked changes. You should upload this as a separate file labeled 'Manuscript'.

If you would like to make changes to your financial disclosure, competing interests statement, or data availability statement, please make these updates within the submission form at the time of resubmission. Guidelines for resubmitting your figure files are available below the reviewer comments at the end of this letter.

As the corresponding author, your ORCID iD is verified in the submission system and will appear in the published article. PLOS supports the use of ORCID, and we encourage all coauthors to register for an ORCID iD and use it as well. Please encourage your coauthors to verify their ORCID iD within the submission system before final acceptance, as unverified ORCID iDs will not appear in the published article. Only the individual author can complete the verification step; PLOS staff cannot verify ORCID iDs on behalf of authors.

We look forward to receiving your revised manuscript.

Kind regards,

Richard James Stanton

Academic Editor

PLOS Pathogens

Donna Neumann

Section Editor

PLOS Pathogens

Sumita Bhaduri-McIntosh

Editor-in-Chief

PLOS Pathogens

orcid.org/0000-0003-2946-9497

Michael Malim

Editor-in-Chief

PLOS Pathogens

orcid.org/0000-0002-7699-2064

**Additional Editor Comments (if provided):**

One of the original reviewers was unable to provide a re-review, so a third reviewer was recruited to comment on whether the original reviews had been addressed. They have confirmed that the original review questions have been met, but raise two additional points - one relates to an immunofluorescence figure and the other to a point for the discussion. These questions are important but fair - the latter can be addressed through text changes, the former may also be explainable through text changes or experimentation.

**Journal Requirements:**

If the reviewer comments include a recommendation to cite specific previously published works, please review and evaluate these publications to determine whether they are relevant and should be cited. There is no requirement to cite these works unless the editor has indicated otherwise.

1) We have noticed that you have uploaded Supporting Information files, but you have not included a list of legends. Please add a full list of legends for your Supporting Information files after the references list.

2) PLOS now requires that authors provide the original uncropped and unadjusted images underlying all blot or gel results reported in a submission’s figures or Supporting Information files. This policy and the journal’s other requirements for blot/gel reporting and figure preparation are described in detail at https://journals.plos.org/plosone/s/figures#loc-blot-and-gel-reporting-requirements and https://journals.plos.org/plosone/s/figures#loc-preparing-figures-from-image-files.

When you submit your revised manuscript, please ensure that your figures adhere fully to these guidelines and provide the original underlying images for all blot or gel data reported in your submission. See the following link for instructions on providing the original image data: https://journals.plos.org/plosone/s/figures#loc-original-images-for-blots-and-gels.

In your cover letter, please note whether your blot/gel image data are in Supporting Information or posted at a public data repository, provide the repository URL if relevant, and provide specific details as to which raw blot/gel images, if any, are not available. Email us at customercare@plos.org if you have any questions.

**Reviewers' Comments:**

Reviewer's Responses to Questions

**Part I - Summary**

Please use this section to discuss strengths/weaknesses of study, novelty/significance, general execution and scholarship.

Reviewer #1: The authors have addressed my previous suggestions.

Reviewer #3: Zhao et al. submitted a revised manuscript reporting novel results showing interactions of the host proteins TRIM5alpha and cyclophilin A with the VP19c subunit of the capsid triplex of herpes simplex virus (HSV) and its orthologs in VZV, HCMV, EBV, and KSHV.

The authors furthermore show, for the first time, that the interferon-inducible TRIM5alpha is a restriction factor for HSV-1, and that this activity requires its Ub ligase activity, the PRY-SPRY domain, and oligomerization. Moreover, they report that VP19c induces NF-kappaB signalling independently and synergistically with TRIM5alpha. In contrast, the other host factor, cyclophilin A, is proviral; however, for HSV-1, unlike other viral infections, this proviral activity does not depend on TRIM5alpha. Based on these data, the authors propose testing whether cyclosporin A, which targets cyclophilin and inhibits neuronal HSV infections, could be useful in treating HSV encephalitis.

A major weakness of this study is that the authors did not address whether TRIM5alpha and cyclophilin A interact only with soluble forms of VP19c and its orthologs, or whether they also interact with VP19c after its incorporation into viral particles. The authors did not conduct any experiments with viral capsids, yet they frame their study in the context of viral capsids as potential targets for pattern recognition receptors (introduction, lines 75-76; Results, lines 196; discussion, lines 315 - 319). On the other hand, the authors missed that the GTPase Mx2 (MxB) has already been shown to limit HIV and HSV infection by restricting viral capsids.

Furthermore, figure 4 does not align with published studies by other groups on the subcellular localization of capsids and capsid proteins like VP19c during HSV-1 infection.

**********

**Part II – Major Issues: Key Experiments Required for Acceptance**

Please use this section to detail the key new experiments or modifications of existing experiments that should be absolutely required to validate study conclusions.

Generally, there should be no more than 3 such required experiments or major modifications for a "Major Revision" recommendation. If more than 3 experiments are necessary to validate the study conclusions, then you are encouraged to recommend "Reject".

Reviewer #1: N/A

Reviewer #3: Overall, the authors have adequately addressed the reviewer's comments on the first submission.

However, the data in Figure 4 is still confusing. It is only briefly described in the results section, and the legend provides little information.

Why is there a signal for VP19C in the mock-condition? Is Mock the 0 h time point? None of the images, including those in the zoom setting, indicate that TRIM5alpha is targeted to or enriched at the nuclear envelope. All images suggest a rather uniform distribution throughout the entire cytoplasm.

Furthermore, the images are inconsistent with the concept that TRIMalpha5 restricts HSV-1 infection. Some cells show a strong VP19c signal, while other cells do not reveal any VP19c signal at all. Furthermore, VP19c is a capsid protein; thus, it should be efficiently targeted to the nucleus (as also described in the introduction), and its accumulation should increase over time in the nucleus and later in the cytoplasm. These data are inconsistent with other groups' reports on the subcellular localization of capsids and capsid proteins during an HSV-1 infection.

Furthermore, these data do not align with the authors' description in the discussion (lines 330-332). It is not true that there are no antibodies directed against HSV-1 capsid proteins and suitable for immunofluorescence microscopy (discussion, line 342). Furthermore, several HSV-1 strains are available in which the capsid proteins have been tagged with autofluorescent proteins, which could be used in cells expressing different versions of TRIMalpha5.

**********

**Part III – Minor Issues: Editorial and Data Presentation Modifications**

Please use this section for editorial suggestions as well as relatively minor modifications of existing data that would enhance clarity.

Reviewer #1: N/A

Reviewer #3: The authors should refer to Mx1 as an established interferon-inducible host protein that has been proven to restrict the functions of viral capsids, and address in their discussion the limitation of their present work as they have not elucidated whether TRIM5alpha limits HSV-1 infection by perturbing capsid functions or just intracellular trafficking of the capsid protein V19c.

**********

PLOS authors have the option to publish the peer review history of their article (what does this mean?). If published, this will include your full peer review and any attached files.

If you choose “no”, your identity will remain anonymous but your review may still be made public.

**Do you want your identity to be public for this peer review?**  For information about this choice, including consent withdrawal, please see our Privacy Policy.

Reviewer #1: No

Reviewer #3: No

[NOTE: If reviewer comments were submitted as an attachment file, they will be attached to this email and accessible via the submission site. Please log into your account, locate the manuscript record, and check for the action link "View Attachments". If this link does not appear, there are no attachment files.]

**Figure resubmission:**

-->While revising your submission, we strongly recommend that you use PLOS’s NAAS tool (https://ngplosjournals.pagemajik.ai/artanalysis) to test your figure files. NAAS can convert your figure files to the TIFF file type and meet basic requirements (such as print size, resolution), or provide you with a report on issues that do not meet our requirements and that NAAS cannot fix.

After uploading your figures to PLOS’s NAAS tool - https://ngplosjournals.pagemajik.ai/artanalysis, NAAS will process the files provided and display the results in the "Uploaded Files" section of the page as the processing is complete. If the uploaded figures meet our requirements (or NAAS is able to fix the files to meet our requirements), the figure will be marked as "fixed" above. If NAAS is unable to fix the files, a red "failed" label will appear above. When NAAS has confirmed that the figure files meet our requirements, please download the file via the download option, and include these NAAS processed figure files when submitting your revised manuscript.

**Reproducibility:**

To enhance the reproducibility of your results, we recommend that authors of applicable studies deposit laboratory protocols in protocols.io, where a protocol can be assigned its own identifier (DOI) such that it can be cited independently in the future. Additionally, PLOS ONE offers an option to publish peer-reviewed clinical study protocols. Read more information on sharing protocols at https://plos.org/protocols?utm_medium=editorial-email&utm_source=authorletters&utm_campaign=protocols

# Author comment: Author response to Decision Letter 2

**DOI:** 10.1371/journal.ppat.1014376.r004

11 Jun 2026

Attachment Submitted filename: Response_to_Reviewers_auresp_2.docx

# Editor report: Decision Letter 2

**DOI:** 10.1371/journal.ppat.1014376.r005

**Authors:** Richard James Stanton, Donna M Neumann

12 Jun 2026

Dear Dr Zhao,

We are pleased to inform you that your manuscript 'Independent proviral and antiviral host factors recognize the same capsid protein in divergent human herpesviruses' has been provisionally accepted for publication in PLOS Pathogens.

Before your manuscript can be formally accepted you will need to complete some formatting changes, which you will receive in a follow up email. A member of our team will be in touch with a set of requests.

Please note that your manuscript will not be scheduled for publication until you have made the required changes, so a swift response is appreciated.

IMPORTANT: The editorial review process is now complete. PLOS will only permit corrections to spelling, formatting or significant scientific errors from this point onwards. Requests for major changes, or any which affect the scientific understanding of your work, will cause delays to the publication date of your manuscript.

Should you, your institution's press office or the journal office choose to press release your paper, you will automatically be opted out of early publication. We ask that you notify us now if you or your institution is planning to press release the article. All press must be co-ordinated with PLOS.

Thank you again for supporting Open Access publishing; we are looking forward to publishing your work in PLOS Pathogens.

Best regards,

Richard James Stanton

Academic Editor

PLOS Pathogens

Donna Neumann

Section Editor

PLOS Pathogens

Sumita Bhaduri-McIntosh

Editor-in-Chief

PLOS Pathogens

orcid.org/0000-0003-2946-9497

Michael Malim

Editor-in-Chief

PLOS Pathogens

orcid.org/0000-0002-7699-2064

***********************************************************

Reviewer Comments (if any, and for reference):

# Editor report: Acceptance letter

**DOI:** 10.1371/journal.ppat.1014376.r006

**Authors:** Richard James Stanton, Donna M Neumann

Dear Dr Zhao,

We are delighted to inform you that your manuscript, "

Independent proviral and antiviral host factors recognize the same capsid protein in divergent human herpesviruses," has been formally accepted for publication in PLOS Pathogens.

We have now passed your article onto the PLOS Production Department who will complete the rest of the pre-publication process. All authors will receive a confirmation email upon publication.

The corresponding author will soon be receiving a typeset proof for review, to ensure errors have not been introduced during production. Please review the PDF proof of your manuscript carefully, as this is the last chance to correct any scientific or type-setting errors. Please note that major changes, or those which affect the scientific understanding of the work, will likely cause delays to the publication date of your manuscript. Note: Proofs for Front Matter articles (Pearls, Reviews, Opinions, etc...) are generated on a different schedule and may not be made available as quickly.

Soon after your final files are uploaded, the early version of your manuscript, if you opted to have an early version of your article, will be published online. The date of the early version will be your article's publication date. The final article will be published to the same URL, and all versions of the paper will be accessible to readers.

For Research Articles, you will receive an invoice from PLOS for your publication fee after your manuscript has reached the completed accept phase. If you receive an email requesting payment before acceptance or for any other service, this may be a phishing scheme. Learn how to identify phishing emails and protect your accounts at https://explore.plos.org/phishing.

Thank you again for supporting open-access publishing; we are looking forward to publishing your work in PLOS Pathogens.

Best regards,

Sumita Bhaduri-McIntosh

Editor-in-Chief

PLOS Pathogens

orcid.org/0000-0003-2946-9497

Michael Malim

Editor-in-Chief

PLOS Pathogens

orcid.org/0000-0002-7699-2064
